# Application strategies of autologous and decellularized nerve grafts: Structural and functional recovery

**DOI:** 10.4103/NRR.NRR-D-25-00607

**Published:** 2025-09-03

**Authors:** Xiaoqi Yang, Nianci Huo, Hui Zhou, Senrui Li, Mengyuan Fang, Nan Zhou

**Affiliations:** 1 Department of Orthopedics, The First Affiliated Hospital of Zhengzhou University, Zhengzhou, Henan Province, China; 2 Department of Ophthalmology, The First Affiliated Hospital of Zhengzhou University, Zhengzhou, Henan Province, China

**Keywords:** allograft, autograft, innervation, mesenchymal stem cells, nerve regeneration, neuroma, peripheral nerves, scar, Schwann cells, stem cells, tissue engineering

## Abstract

Autologous nerve transplantation is currently recognized as the gold standard for treating severe peripheral nerve injuries in clinical practice. However, challenges such as a limited supply of donors, complications in the donor area, and the formation of neuromas necessitate the optimization of existing transplantation strategies. Additionally, the development of new and promising repair methods is a critical issue in the field of peripheral nerve research. The purpose of this article is to compare the advantages and disadvantages of autologous, allogeneic, decellularized nerve grafts, and cell-composite graft, as well as to summarize the differences in their prognostic factors and associated adverse events. The length, diameter, polarity, and sensory or motor origin of autografts all influence axonal regeneration. While pre-denaturation treatment can accelerate early regeneration, long-term functional outcomes of autografts do not show significant differences compared with fresh autologous grafts. For decellularized nerve grafts, defect length is identified as an independent risk factor, and the internal microenvironment (delayed angiogenesis, Schwann cell senescence, and reduced T-cell infiltration) is considered a key factor limiting long-segment regeneration. Additionally, the decellularization process (whether chemical, physical, or supercritical CO_2_) affects the integrity of the extracellular matrix and the presence of immune residuals, which directly impacts axonal guidance and host integration. Common adverse events following autograft transplantation include donor site numbness, neuromas, and scarring. In contrast, adverse events associated with decellularized nerve graft transplantation may present as inflammatory reactions, excessive scar proliferation, and misalignment or reconnection of regenerating axons, which can lead to sensory–motor cross-innervation. To mitigate these issues, combining decellularized nerve grafts with autologous Schwann cells, mesenchymal stem cells, or induced pluripotent stem cell–derived cells may help bridge the gap with autografts. However, the fact that structural recovery does not necessarily lead to functional recovery needs further clarification. Future research should establish large animal models to replicate the limits of human regenerative capacity, use gene editing to enhance the phenotype and microenvironment of transplanted cells, and develop a mild combined decellularization process that maximizes the preservation of natural nerve grafts. Through multidimensional optimization, decellularized nerve grafts have the potential to ultimately replace autograft transplantation, enabling precise repair of individualized, long-segment, and complex nerve defects.

## Introduction

The peripheral nervous system (PNS) is characterized by its extensive distribution throughout the human body, with nerve fibers extending to various anatomical regions (Murtazina et al., 2023). This widespread network is essential for the proper functioning of numerous physiological processes, thereby playing a vital role in maintaining overall homeostasis. However, this extensive anatomical distribution also renders peripheral nerves highly susceptible to various forms of injury, mainly including physical trauma and iatrogenic damage, particularly those in the face and limbs due to their superficial location (Hara et al., 2021; Robinson et al., 2022). Among these injuries, nerves in the upper extremities, particularly those at or distal to the wrist, are most susceptible to damage. Such injuries can severely disrupt the normal physiological functions of the body, ultimately leading to sensory deficits or motor dysfunction in affected individuals (Barneset al., 2022; Lopes et al., 2022). It is worth noting that the incidence of such injuries is steadily increasing and is significantly higher in males (Li et al., 2021b). This is particularly concerning, given that our limbs are almost entirely involved in all daily activities.

Although peripheral nerve injuries are generally less life-threatening than central nervous system (CNS) injuries, their consequences can be profoundly serious and long-lasting, remarkably impacting the quality of life of patients. Compared with the central nervous system, the peripheral nervous system exhibits a markedly greater regenerative capacity (Adidharmaet al., 2022; Cooke et al., 2022). However, despite the robust regenerative potential observed in animal models, achieving complete restoration of sensory and motor functions in humans remains a considerable challenge (Maugeri et al., 2021; Juckett et al., 2022). In patients suffering from severe peripheral nerve injuries, the functional recovery frequently fails to meet clinical expectations, leaving various complications (Guo et al., 2024a, b; You et al., 2025; Zheng et al., 2025). These include continuous numbness and pain in the affected regions, decreased limb mobility, and even major impairment, which can cause great misery and place a massive strain on the affected persons (Zhang et al., 2022; Wong et al., 2024).

Current clinical management strategies for peripheral nerve injuries encompass a range of therapeutic modalities, including pharmacotherapy, physical therapy, rehabilitation therapy, and surgical intervention (Baradaran et al., 2021; Maeng et al., 2022; Ni et al., 2023). Among them, surgical repair is widely regarded as the optimal clinical strategy for severe nerve injuries and nerve transections. Specifically, autologous sensory nerve grafting has long been recognized as the gold standard for treating long-segment peripheral nerve defects (Xu et al., 2024a). The primary objective of neurosurgical treatment for peripheral nerve injuries is to restore nerve continuity, minimize axonal loss at the site of injury, and facilitate the regeneration of sensory, motor, and autonomic axons (Bellaire et al., 2021; Singh et al., 2022). In clinical nerve transplantation, autologous pure sensory nerves, such as the sural nerve, are commonly selected as donor nerves to bridge transected peripheral nerves (Poppler et al., 2015; Piedra et al., 2023). The use of autografts mitigates the risk of immune rejection. However, this approach often necessitates a trade-off between sensory function in donor sites and the prioritization of motor function recovery in the recipient site (Bamba et al., 2021; Raizman et al., 2023). By re-establishing motor innervation to damaged muscle tissues, this strategy aims to improve the patient’s prognosis and reduce the risk of long-term disability (Hu et al., 2021). Autologous nerve grafting, the gold standard in clinical practice, has lots of drawbacks, including donor-site morbidity resulting from the harvest procedure, limited availability of nerve length for grafting, and the potential for neuroma formation at the proximal end of the graft (Geissler et al., 2019; Lans et al., 2023; Adidharma et al., 2024). For nerve defects larger than 3 cm, allogeneic nerve grafts are typically considered (Kornfeld et al., 2021). However, the use of allogeneic nerve grafts is associated with several limitations, including the risk of immune rejection necessitating immunosuppressive therapy and the potential for postoperative complications (Imet al., 2022; Saffari et al., 2024). Existing transplant treatments are unable to achieve excellent functional recovery and cannot overcome drawbacks, such as difficulties in obtaining donor tissue and immune responses (Bittner et al., 2022). Therefore, optimizing transplantation strategies and exploring promising alternative approaches are key areas for development in the field of peripheral nerve regeneration.

Grafts are key to nerve transplantation, and their properties significantly influence post-transplantation survival and regenerative capacity. Therefore, optimizing the graft itself is a promising strategy. Factors, such as the size, length, polarity, and sensory or motor origin of the graft have been studied by many scientists for their effects on final functional recovery (Nichols et al., 2004; Cheah et al., 2017; Lee et al., 2021; Zhu et al., 2023). Contrary to common belief, the size of donor and recipient nerves appears to influence final neural regeneration outcomes only within a certain range (Brenner et al., 2006). That is, matching the graft cross-section via cable grafting does not necessarily yield better regenerative effects than single-nerve grafting. Although many surgeons habitually believe that reverse nerve transplantation yields better results because the reverse nerve basement membrane tube is smaller and more suitable for proper axon innervation and regeneration, recent studies have shown that nerve polarity has no statistical difference on the final functional recovery (Kim et al., 2020; Lee et al., 2024). It is necessary to analyze and discuss these properties of the graft. The patient’s health condition at the time of surgery cannot be changed, but we can try most to optimize the graft itself so that it is in the best condition for regeneration.

Decellularized nerve grafts and nerve conduits offer a promising approach for developing alternatives to natural nerve grafts. They provide a natural scaffold suitable for axonal regeneration and can be combined with processed cell components that promote nerve regeneration, thereby facilitating superior functional recovery in damaged nerves (Contreras et al., 2022; Mehta et al., 2024; Zhu et al., 2025). Schwann cells and various stem cells have gained lots of attention due to their ability to express neurotrophic cytokines and support axonal growth (Wang et al., 2022). With the advancement of tissue engineering and material synthesis techniques, there has been a significant increase in the number of reports highlighting the emerging role of cell-based support therapies in peripheral nerve repair (de Assis et al., 2023; Fan et al., 2025). Commercial decellularized nerve grafts have been used in clinical treatment for nearly 20 years, and new breakthroughs in cell therapy has also increasingly been reported (Kasper et al., 2020; Ferreira et al., 2025). The combination of the two is considered to be a hot topic for future research in the field of peripheral nerve regeneration and tissue engineering. These treatment methods are expected to overcome the limitations of traditional ones and improve treatment outcomes for patients with severe and long-term neurological damage.

**Figure 1:**
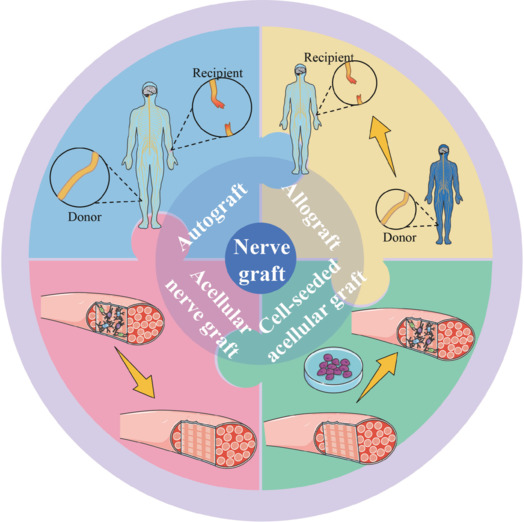
Nerve grafting and other graft-based therapies for nerve defects.

**Figure 2:**
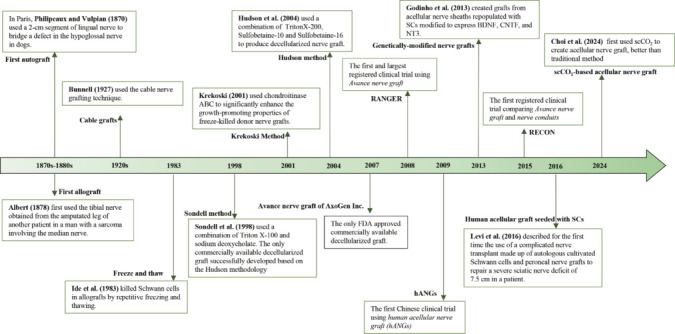
Timeline illustrating breakthroughs and key research in nerve grafting and decellularized grafts. BDNF: Brain-derived neurotrophic factor; CNTF: ciliary neurotrophic factor; FDA: The Food and Drug Administration; hANCs: human acellular nerve graft; NT3: neurotrophin-3; scCO_2_: supercritical carbon dioxide; SCs: Schwann cells.

In summary, this review provides a comprehensive overview of the historical trajectory and future directions of research related to nerve transplantation (**Figure 1**). We have systematically reviewed the development and significant advancements in nerve transplantation and its alternative therapies, with a primary focus on autologous nerve transplantation, allogeneic nerve transplantation, decellularized nerve grafts, and the integration of cellular components with decellularized nerve grafts (**Figure 2**). It is important to note that nerve conduits have not been included in this review, as their study is more closely aligned with the field of material science. Drawing on previous research findings and clinical experience, we have analyzed the factors that may influence functional recovery following nerve transplantation (**Additional Table 1**). Furthermore, we discuss about the safety and biocompatibility of decellularized nerve grafts and their clinical trials and translations. Lastly, we present our perspective on several key issues related to nerve graft research. We hope that this review will contribute to the field by providing novel insights and ideas for the advancement of peripheral nerve transplantation therapy.

**Additional Table 1:** Main therapies for nerve defects using nerve grafts

	Autograft	Allograft	Acellular nerve graft	Decellularized grafts seeded with cells
Disadvantages	(1) Limited fetch length; (2) Donor site morbidity; (3) Prolonged surgery for nerve harvesting	(1) Immune rejection; (2) Immunosuppressive drug adverse effects; (3) Ethical issues	(1) Decellularization is time-consuming and costly; (2) Lack of some cellular components prolongs regeneration time; (3) Low vascularization	(1) Expanding and inducing cells *in vitro* is time-consuming and costly; (2) Harvesting may be invasive (e.g., bone marrow mesenchymal stem cells)
Advantages	(1) Low or no immune rejection; (2) Better recovery	(1) Do not affect nerve function in the donor site; (2) Unlimited length, size, and type of nerves	(1) Unlimited length, size, and type of nerves; (2) Do not affect nerve function in the donor site; (3) Can be taken between species; (4) Preserve 3D scaffolding while minimizing immunogenicity, even without immune rejection	(1) Implanted cells can be easily modified through techniques such as gene editing; (2) Targeted addition of specific cells or nutrient-supporting factors to accelerate axonal regeneration
Influential factors	(1) Graft length, size, motor or sensory origin, polarity, pre-degeneration treatment; (2) Donor status, including health and age		(1) Decellularization method; (2) Compatibility of decellularized tissue scaffolds; (3) Length of nerve defects; (4) Donor status, including health, and age	(1) Decellularization method; (2) Implanted cells; (3) Cell harvesting and *in vitro* processing
Adverse effects	(1) Neuroma; (2) Nerve scarring; (3) Donor site morbidity; (4) Pain	(1) Immune rejection; (2) Neuroma; (3) Nerve scarring; (4) Pain	(1) Inflammatory response; (2) Neuroma	(1) Inflammatory response; (2) Neuroma
Prognosis	Clinical gold standard	No significant difference from autologous nerve grafts in the clinic	In the clinic, as good as autografts for defects smaller than 3 cm, but not for long, large-diameter nerves	In the clinic, the effective regeneration length is greater than that of decellularized grafts alone

## Search Strategy

We conducted a search of the PubMed and Web of Science databases between February 2025 and June 2025 using the following search terms: “Nerve grafting,” “Nerve grafts,” “Peripheral nerve regeneration,” “Anatomy of peripheral nerve,” “Axonal regeneration,” “Wallerian degeneration,” “Nerve autograft,” “Nerve allograft,” “Acellular nerve graft,” “Decellularized nerve graft,” “Processed nerve graft,” “Stem cells,” “Schwann cells,” “Mesenchymal stem cells,” “Sensory nerve,” “Motor nerve,” “Mixed nerve,” “Gene editing,” “Vascularization,” “Nerve scarring,” “Neuroma,” “Disordered regeneration,” “Immune rejection,” “Biocompatibility,” “Decellularization strategy,” “Peripheral nerve,” “PNS,” “Nerve gap,” “Nerve defect,” “Nerve length,” “Nerve polarity,” “Nerve orientation,” “Induced pluripotent stem cells,” “Neural stem cells,” “Neural progenitor cells,” and “Adverse effects.” We used various combinations of these keywords to perform searches without any date restrictions. The literature resulting from these search term combinations was further screened based on titles and abstracts, and full texts were reviewed to ensure comprehensive coverage of the topic. This review concentrates on the therapeutic applications of natural nerve grafts and their decellularized counterparts, examining the various factors that may influence their efficacy. Additionally, we have incorporated numerous studies related to alternative nerve graft materials to facilitate comparative analysis and to explore the unique advantages of natural nerve structures.

For the retrieval of clinical research on acellular nerve grafts, the International Clinical Trials Registry Platform (ICTRP) and ClinicalTrials.gov (CTG) were utilized with the following search terms: “Acellular nerve graft,” “Processed nerve graft,” “AVANCE,” “Acellular nerve,” and “Decellularized nerve.” We included clinical trials evaluating the effects of decellularized nerve grafts on nerve repair in this study. Studies with the status of “not yet recruited” and those not focused on peripheral nerve regeneration and repair after nerve injury were excluded.

## Overview of Peripheral Nerve Anatomy and Regeneration

### Nerve anatomy

**Figure 3:**
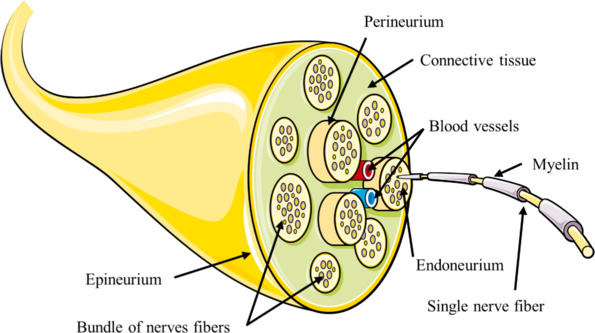
Anatomy of a peripheral nerve.

The somata of peripheral neurons are localized within the dorsal root ganglia for sensory neurons and the anterior horn of the spinal cord for motor neurons (**Figure 3**). From these locations, their axonal processes extend peripherally (Meltzer et al., 2021). These axonal structures, as extensions of the neurons, facilitate communication between the central nervous system and peripheral organs. The architecture of peripheral nerves is distinguished by a tripartite connective tissue sheath, which is hierarchically organized from the innermost to the outermost layer as the endoneurium, perineurium, and epineurium (Iwanaga et al., 2022; McLeod et al., 2024). Collectively, these concentric layers form an intricate protective and supportive matrix that encapsulates the nerve fibers. This structural arrangement is essential for safeguarding the integrity of nerve conduction and ensuring the efficient propagation of neural impulses within the complex *in vivo* environment (Liu et al., 2019). The fundamental structural and functional unit of the peripheral nerve is the nerve fiber, which consists of the axon and its associated specialized neuroglial cells, specifically the Schwann cells (Mathis et al., 2025). Schwann cells play a crucial role in supporting and maintaining the axonal integrity (Han et al., 2025). There are also endothelial cells, macrophages, and adipocytes distributed in the peripheral regions of the nerve, contributing to the overall structural framework and metabolic homeostasis of the nerve tissue (Huang et al., 2024; Choi et al., 2025; Li et al., 2025; Sprenger et al., 2025). In addition to the aforementioned cellular parts, the peripheral nerve contains an extracellular matrix scaffold, a dense network of nutritive blood arteries, and lymphatic structures that perform metabolic activities (Brown et al., 2022; Yu et al., 2023). These elements collectively constitute the intricate and multifaceted architecture of the peripheral nerve.

### Axon regeneration and Wallerian degeneration

When the connection between neurons and their target organs is disrupted or severed, the neurons undergo a phenotypic transformation and enter a regenerative state marked by remarable changes in gene expression (Supra et al., 2023). This adaptive response of peripheral neurons is essential for initiating and promoting nerve regeneration. At the site of injury, large amounts of extracellular sodium and calcium ions rush into the damaged axoplasm, thereby inducing action potentials. It is currently widely believed that the burst of action potentials triggered by the injury site and the disruption of axonal transport are the key triggering factors for neuronal regeneration after axonal transection (Mahar et al., 2018; Gordon et al., 2024a). At the same time, the injury-induced interruption of normal axoplasmic transport generates negative feedback signals that alert the soma to the disconnection.

**Figure 4:**
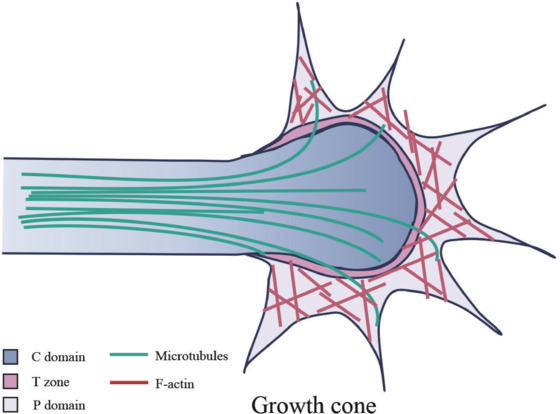
Schematic structure of a growth cone.

Severed axons undergo a transformation, forming highly active terminals called growth cones (Nozumiet al., 2025; **Figure 4**). The growth cone is composed of three main functionally distinct regions: the central structural domain (microtubule-rich C-region), the transition region (T-region), and the peripheral structural domain (actin-rich P-region) (Leite et al., 2021). These areas work together to promote the growth cone to move forward through a sequence of steps involving protrusion, engorgement, and consolidation (Bradke et al., 2012). The growth direction of the growth cone is guided by gradients of neurotrophic factors and pro-neurotrophic factors, which are mainly produced by non-neuronal cells (Shim et al., 2010). The growth cone continuously checks its microenvironment and precisely regulates the extension of the regenerating axon. However, when growth cones fail to reach the distal stump, the innervation to the target organ will not be successfully established, leading to the poor functional recovery and other adverse effects.

Within the first 24 hours after nerve injury, distal nerves undergo Wallerian degeneration and proximal axons simultaneously begin to sprout and regenerate (Zhang et al., 2021a). The distal segment of the axon undergoes rapid degeneration and breakdown of the axon and myelin sheath, and this process proceeds in a proximal-to-distal direction. This activation is distinguished by morphological changes such as larger nuclei and cytoplasm, as well as faster mitosis (Esposito et al., 2002). Depending on the severity of the injury, similar changes may also occur at varying distances proximal to the injury site (Kerns et al., 2021; Liu et al., 2023b). Schwann cells respond rapidly to damage, activating within 24 hours. Within the distal nerve segment, cells differentiate into a repair phenotype and recruit macrophages to the injury site. Together, they phagocytose and remove the degenerated axonal and myelin fragments, thereby creating a favorable environment for axon regeneration (Wu et al., 2023). The growing Schwann cells then create Büngner bands, which act as important guiding structures for the formation of regenerated axons (Pestronk et al., 2023). This process may last from 1 week to several months, depending on the complexity of the injury and the individual’s physiological response.

## Nerve Grafts

The integrity of neural structures constitutes a prerequisite for the successful regeneration of axons. In cases of a mild peripheral nerve injury, direct suture of the severed ends is generally feasible, provided that minimal tension is ensured (Brage et al., 2022). However, in severe nerve injuries, particularly following peripheral nerve transection, the nerve undergoes a reduction in length, which complicates the process of directly suturing the severed ends. Additionally, there exists an inverse relationship between nerve tension and blood flow (McQuillan et al., 2024). When nerve tension is too high, blood supply to the nerves is obstructed, leading to poor recovery results. In instances where nerve tension cannot be adequately managed, nerve grafting is frequently selected as a viable method for repairing transected nerves. In 1870, Philipeaux and Vulpian first used a 2-cm segment of lingual nerve to bridge a defect in the hypoglossal nerve in dogs (Irisarri et al., 2024). In 1878, Albert first used the tibial nerve obtained from the amputated leg of another patient in a man with a sarcoma involving the median nerve. Their pioneering efforts were truly groundbreaking, and it was during this period that the scientific community began to recognize the potential for physical repair of damaged nerves (Irisarri et al., 2024). The procedures for nerve grafting treatment are diverse, with the choice primarily depending on the specific clinical situations. Autologous nerve grafting is typically used for those who are in good health, have a tiny nerve gap, and can reconstruct the damaged nerve with direct grafting while cleaning it up (Beris et al., 2019; Xu et al., 2024a). This method is widely regarded as the gold standard for nerve grafting procedures, consistently demonstrating superior recovery outcomes compared with other nerve grafting treatments. However, the limitations of autologous nerve grafting are also pronounced. These factors include the finite availability of donor nerves, the restricted length of nerves that can be harvested, and the potential for excessive autologous nerve harvesting, which may lead to additional donor-site morbidity and the occurrence of symptomatic neuroma (Berger et al., 1978). Allogeneic nerve grafts refer to the transplantation of donor nerves into recipients of the same species (Mackinnon et al., 2001; Jacobs et al., 2024). Cadavers serve as the primary source of allogeneic nerve grafts in clinical treatment, providing nerves of various sizes, lengths, and types. This approach effectively overcomes the limitations associated with autologous nerve grafts. However, the transplantation of nerves from another individual may elicit immune rejection at the grafted site or even systemic immune responses, thereby adversely affecting graft survival and regenerative outcomes (Roballoet al., 2022). Consequently, recipients have to take immunosuppressive drugs for an extended period, potentially for life. This long-term medication regimen is often accompanied by significant adverse drug reactions and increased morbidity (Roberts et al., 2021). Despite the rapid advancement of nerve conduit materials designed for pro-regenerative repair, natural nerve grafts remain the predominant choice in clinical treatment (Yu et al., 2024). Therefore, in this section, we will introduce autologous nerve grafts and allogeneic nerve grafts and discuss in detail some of the factors that may have influenced the outcome of regeneration while using nerve grafts in preclinical studies.

### Autografts

Autologous nerve grafts are typically sourced from pure sensory nerves with superficial distributions. The most commonly utilized autologous nerve graft in clinical practice is the peroneal nerve. Other sources of autologous grafts include the anterior branch of the medial cutaneous nerve of the forearm and the radial sensory nerve of the wrist (Stang et al., 2013). The accessibility of these nerves and the minimal impact of their harvest on the motor function of the donor site render them suitable for facilitating the successful restoration of critical motor functions in vital areas.

Autologous nerve grafting, recognized as the treatment yielding the most favorable clinical recovery outcomes, presents several significant advantages over other grafting methods (Sabongiet al., 2014). The utilization of autologous nerve grafts effectively reduces the risk of severe immune rejection. As the harvested nerve originates from the patient’s own body, it is less likely to provoke a robust immune response, which is crucial for maintaining the integrity of the nerve structure and facilitating successful nerve regeneration and repair. Also, autologous nerve grafts demonstrate excellent biocompatibility, offering a well-matched neural scaffold composed of basement membrane tubes and homologous extracellular matrix components for nerve repair (Sánchez et al., 2017). This scaffold supports axonal outgrowth. Additionally, these grafts contain nutrient-supporting cells, such as vascular endothelial cells and Schwann cells, and neurotrophic factors (Yi et al., 2019). Vascular endothelial cells facilitate revascularization, while Schwann cells provide structural support and secrete growth factors and cytokines that enhance axonal elongation and myelination. These actions collectively promote successful nerve regeneration and functional recovery. Further, although the original microvasculature in the grafted nerves is interrupted during the grafting process, functional blood flow is rapidly reconstructed within a few days by anastomosis or spontaneous end-to-end repair of the existing vascular system, which occurs in the tissue bed and at the ends of the reconnected nerves. In addition, these grafts undergo functional and structural remodeling over the next few weeks, including Wallerian degeneration, which mimics the changes that occur after injury to the distal end of a damaged nerve, which in turn induces the initiation of a regenerative repair process between the donor and recipient nerves (Navarro et al., 2009).

Autografts are the gold standard for nerve transplantation and the most appropriate control group in animal experiments, and they possess better functional recovery results than other modalities. However, autografts have significant disadvantages. First, the harvesting of autologous nerve grafts necessitates an additional surgical procedure. The acquisition of donor nerves increases the risk of injury and morbidity at the donor site, including potential loss of function at the donor site. Additionally, various adverse complications may arise, such as nerve scarring and the formation of neuromas. Second, the availability of suitable donor nerves is often limited, particularly when repairing large nerve defects. The diameter of the harvested donor nerves is typically smaller than the cross-sectional area of the nerve requiring repair. This discrepancy may adversely impact the final results of functional recovery.

### Allografts

When the gap of nerve injury is too large, exceeding the appropriate length that can be provided by the autologous nerve, or when autologous harvesting considerably affects the function of the harvested area, the autologous nerve is usually unable to fulfill the needs of the graft. This has encouraged the search for alternative reconstruction methods for extensive nerve injuries. The utilization of allografts for the repair of extensive nerve defects provides a potential solution to maintain the continuity of peripheral nerves, thereby facilitating regenerative repair.

Allografts are typically sourced from cadavers, thereby circumventing many of the limitations associated with autologous nerve grafts (Isaacs et al., 2024). They can be tailored to repair various types of nerves, including motor, sensory, and mixed nerves, according to the specific requirements of the recipient. Furthermore, allografts are not constrained by limitations in length or diameter, thus enabling highly specific nerve grafting treatments. However, allogeneic transplantation faces a common problem in the field of organ transplantation - immune rejection. This reaction is thought to result in significant infiltration of inflammatory cells and proliferation of fibrous scar tissue, which can hinder the recovery of regenerating nerves. Schwann cells are the main cellular component in peripheral nerves and only survive in the grafts when immunosuppressive agents are administered and are considered to be one of the main sources of immunogenicity, other cells include endothelial cells and macrophages (Rovak et al., 2005). Immune responses against major histocompatibility have limited their widespread use and efficacy in the clinic. Recently, it has been found that substantial regeneration and functional recovery can occur in allogeneic grafts without the use of immunosuppressive agents, and that the early infiltration of immune cells is slower in allogeneic grafts than in autografts (Liu et al., 2012b).

Currently, immune rejection of grafts can be suppressed in two ways: by reducing the immunogenicity of the donor tissue or by suppressing the immune response to the recipient. A variety of methods have been reported to suppress the immunogenicity of nerve grafts, including lyophilization, radiation, and storage in Cialit solution (Ray et al., 2011; Davis et al., 2023). Although successful experimental and clinical allografts have been reported, there have been many failures. Therefore, there is still a long way to go before the method shows great potential in the clinic. In addition to this, allografts require long-term use of expensive immunosuppressive drugs after transplantation, which are associated with considerable clinical morbidity. In this case, the use of allografts in peripheral nerve repair in actual clinical practice is very rare and limited to the repair of the most severe nerve injuries with a considerable length of nerve defects.

### Factors affecting nerve grafts

While nerve grafts are crucial in repairing transected nerve injuries, patients who have undergone nerve grafting often experience prolonged functional recovery, and the outcomes often fail to meet the expectations. In response to this phenomenon, many clinicians and researchers have focused on the intrinsic properties of nerve grafts themselves (Millesiet al., 2006). Fawcett et al. (1986) reported that as a nerve graft, it must have the following characteristics: (a) regenerating axons that can grow into and pass through the graft to the distal site; (b) axons that can mature after passing through the graft to a point where they achieve a normal diameter, become myelinated, and conduct action potentials; (c) it is not antigenic; (d) it is able to acquire blood flow relatively quickly; and (e) the regenerating axons are arranged in the graft in an orderly fashion to ensure that they accurately reach the target tissues and organs. How we use the nerve grafts will have a critical impact on their recovery outcome. By performing proper interventions on grafts, researchers have explored various methods to enhance the effectiveness and rate of regeneration following nerve grafting.

**Figure 5:**
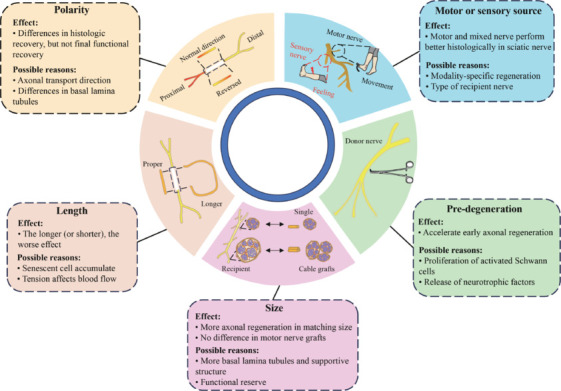
Factors affecting the effectiveness of nerve grafting.

Surgeons often consider the following factors when fixing nerve grafts in patients with nerve injuries, including the type of nerve injury, the time since the injury, the gap size, the diameter of the nerve, and the patient’s individual treatment choices, which leads to the actual grafting process and choices often more complex. In reviewing and summarizing the studies related to nerve transplantation, we noticed some interesting but controversial research projects that respectively discussed the different traits of nerve grafts, including the diameter, sensory or motor origin, polarity, length, and pre-degeneration treatment of grafts (Spector et al., 1991; Ansselinet al., 1993; Dubuisson et al., 1997; Nichols et al., 2004; Brenner et al., 2006; Kawamura et al., 2010; Hoben et al., 2018; Lee et al., 2021; **Figure 5**). The impact of these properties of nerve grafts on axonal regeneration and functional recovery following nerve transplantation remains a subject of ongoing debate (Weber et al., 2023). The findings of this animal studies are expected to provide some reference for nerve grafting therapy in patients to improve the nerve regeneration process.

#### Diameter or size of grafts

**Figure 6:**
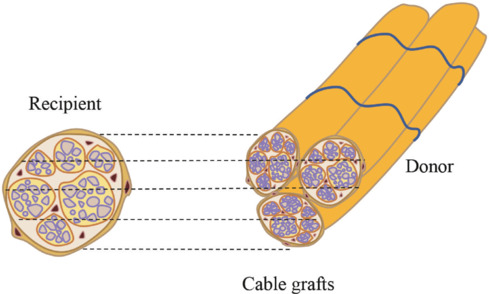
Assembly of cables grafts.

One obvious challenge with autologous nerve transplants is that the grafts used are often small-diameter sensory nerves, while the injured nerves that need repair are frequently mixed or motor nerves with larger diameters. Due to the size differences between the donor and recipient nerves, surgeons find it difficult to satisfactorily match and suture the separated parts of the nerves, which is often regarded as a primary factor influencing nerve function recovery (Isaacs et al., 2014b). But, is the diameter of the nerve graft truly important for nerve regeneration and functional recovery? Currently, there appear to be no clinical studies directly evaluating the effects of different nerve transplant cross-sectional areas (Pover et al., 1989; Zhu et al., 2023). Some investigations have found that the inner diameter of artificial neural conduits has a substantial impact on nerve regeneration. For example, the use of collagen conduits with an inner diameter of 1.5 mm, which is considerably closer to the diameter of the sciatic nerve in rats, resulted in a superior restoration of motor function compared with collagen conduits with a diameter of 2.0 mm (Giusti et al., 2014). To match the size of the donor and recipient nerves, surgeons have devised a nerve grafting strategy known as “Cable graft” (Irisarri et al., 2024). This procedure involves suturing or gluing together several pieces of less significant nerve tissue to create a cable-like structure that fits the diameter of the injured nerve, thereby providing an effective route for nerve regeneration (**Figure 6**). For example, in the repair of facial nerve or brachial plexus injuries, a cable-like graft is often utilized to bridge the gap when there is a wide distance between the proximal and distal ends of the nerve (Humphrey et al., 2008). In a clinical study, 34 patients had their facial nerves repaired via direct end-to-end anastomosis (Spector et al., 1991). At rest, these patients exhibited excellent facial symmetry and muscle tone. Most of them regained voluntary movement, although three showed severe joint band movement. Among the 56 patients with facial nerve injuries who underwent cable grafting, they demonstrated excellent facial symmetry and muscle tone at rest, despite incomplete recovery of voluntary movements, with joint band movements being more common (63%). Furthermore, a study of facial nerve healing in rats using cable grafting suggested that axonal regrowth and function were partially restored (Hohman et al., 2014). These findings support the favorable effects of cable grafts on nerve repair; however, they are not equivalent to direct end-to-end nerve suturing.

However, Brenner et al. (2006) discovered that the quantity of cable grafts made up of donor motor nerves had no effects on regeneration during motor nerve transplantation in rats. These findings are noteworthy because surgeons routinely bundle smaller donor nerves to achieve better size matching when treating large peripheral nerve defects. The findings in animals provide surgeons with new insights and perspectives, and prompt us to question whether the sacrifice of additional nerve tissue to address differences in anastomotic surface size is truly justified. Specifically, does this repair strategy promote the overall outcome of functional recovery? Also, when several nerves are united to form a bundle, their center component may not be in contact with the recipient nerve, thus minimizing graft survival. In addition, microsurgical procedures make it difficult to achieve the same fine sutures in grafts made of numerous cables as in single nerves, potentially leading to failure of axonal regeneration near the distal end or even disordered axonal regeneration at the junction (Prasad et al., 2018).

Cui et al. (2015) investigated the connection between the thickness and function of peripheral nerves in greater detail. They transected the median nerve along its long axis in specific proportions and separated it into groups based on the ratio of the resected section: 1/8, 1/4, 1/3, 1/2, and 2/3. At any time point, the number of anterior horn alpha motor neurons in the experimental and control groups did not differ significantly. Limb edema, deformity, and claudication recovered within 2 weeks postoperatively in the groups with resected areas of less than or equal to 1/3, while the other groups did not experience relief until 4 months postoperatively. The maximal tonic contraction of the muscle decreased as the proportion of the nerve trunk that was cut increased, though not in a strictly proportional manner. Variations in the experimental data within the 1/3 group were less pronounced compared to those in the 1/2 and 2/3 groups. This observation was attributed to the compensatory mechanisms activated by the organism following tissue damage. Based on the findings of this investigation, Lu et al. (2024) concluded that when the fraction of ulnar nerve injury does not exceed 1/3 of its diameter, the negative consequences of the injury are reversible and do not result in permanent functional impairment. They proposed the concept of nerve functional reserve, which refers to the compensatory capacity of a damaged nerve to maintain normal physiological function, quantifiable as the nerve’s functional reserve. In this study, the functional reserve of the rat ulnar nerve was determined to be 1/3 of its trunk diameter; if the degree of injury exceeded this functional reserve, the nerve’s compensatory capacity would be compromised, resulting in permanent damage to nerve function. Although the specific mechanism of neural functional reserve has yet to be elucidated, the phenomenon of “multiple regeneration” following nerve injury may help explain this observation (Peng et al., 2012). An injured axon in the proximal stump of a damaged nerve can regenerate and support three or four lateral shoots at the same time. Gangadharan et al. (2022) found the uninjured peroneal nerve is capable of sprouting lateral branches that follow the original trajectory of the injured tibial nerve, thereby reinnervating the target organ and partially restoring its function. Studies in rhesus monkeys and rats have revealed that one proximal stump can be utilized to heal two distal injured nerves (Zhao et al., 2007; Zhang et al., 2011). In clinical transplantation, it may be possible to take advantage of the nerves’ functional reserve capacity and, instead of using sensory nerves, extract the appropriate amount of nerve tissue from motor or mixed nerves to aid in the recovery of motor function. Of course, all of these require additional research.

#### Polarity of the graft

**Figure 7:**
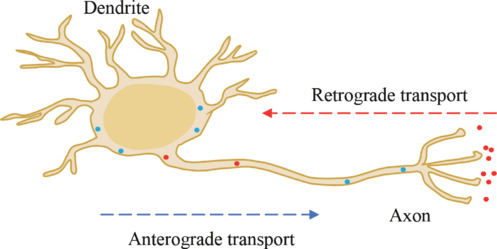
Schematic diagram of axonal transport.

Many scientists believe that the transport of substances within peripheral nerve axons is directional, with substances such as neurotransmitters, proteins, and vesicles moving cis-directionally (from the soma to the axon terminals), whereas some neurotrophic factors such as nerve growth factors (NGFs), exogenous viruses, and toxins moving retrogradely (from the axon terminals to the soma) (Tomé et al., 2024; **Figure 7**). This bidirectional transport pattern is essential for maintaining proper neuronal function, signaling, and damage repair. As a result, some people believe that when performing nerve transplantations, the fresh nerve graft and nerve stump correspondence should be done well to maintain the nerve graft’s normal orientation, i.e., the proximal ends of the donor and recipient nerves match each other, and the distal portions of the nerves match each other.

Currently, during clinical nerve repair, many surgeons consciously reverse the orientation of nerve grafts to improve nerve regeneration by reducing the potential for misrouting due to branching (Lee et al., 2024). Ansselin and Davey (1993) demonstrated this in a rat sciatic nerve model, revealing that reversed autografts have a greater axon count and better conduction velocities than normally oriented autografts, providing a theoretical basis for this approach. They propose that most nerve trunks branch during growth and development, resulting in alterations to the basal lamina tubules in peripheral nerves. When peripheral nerve grafts are oriented in the normal direction, the number of regenerated axons in the distal region of the grafts decreases due to branching into other lamina tubules during the process. In contrast, reversed grafts can reduce axon loss because the lamina tubules in this direction will ultimately converge.

Roberts et al. (2017) conducted a systematic review and analysis of research articles published prior to 2015 that investigated the impact of nerve graft orientation on the regenerative recovery of recipient nerves. Among the six articles that met the criteria for inclusion in their study, none of the animal studies demonstrated significant differences between normally oriented grafts and reverse grafts. Several other researchers have explored this subject over the past decade, with the majority arriving at the same conclusion: nerve polarity has no meaningful effect on final functional recovery (Afshari et al., 2018; Kim et al., 2020). Interestingly, a study reported in 2021 using mice did not evaluate the level of newborn axons based on the number of axons in the nerve cross-section; instead, the authors optimized the method to stain the whole nerve (Lee et al., 2021). They found that normally oriented autologous nerve grafts promote axon regeneration and prevent neurogenic muscular atrophy more effectively than reverse autologous grafts. Additionally, the pattern of axon regeneration differs depending on the orientation of the transplanted nerve. However, despite the fact that regularly oriented grafts exhibited better histologic-morphologic healing outcomes, there was no significant difference in final functional recovery. Currently, the results of most investigations on the polarity of nerve grafts support the idea that polarity has no meaningful effect on the functional recovery of nerves. It is worth noting that the methods and results used to assess nerve regeneration and functional recovery vary greatly between published studies (Ronchi et al., 2019, 2023), and there does not always appear to be consistency between histologic and functional regeneration of peripheral nerves, both of which significantly impact the validity of experimental conclusions. As a result, existing evaluation tools must be optimized, and new assays should be investigated to comprehensively and systematically assess regeneration outcomes following nerve transplantation.

#### Sensory or motor nerve sources

In modern clinical practice, the restoration of critical motor capabilities often comes at the expense of less important sensory functions. Despite the increasing sophistication of microsurgical procedures, recovery of motor function following autologous nerve grafting is frequently unsatisfactory. The distinctions between sensory and motor nerves have been proposed as the primary cause of this poor recovery. Nichols et al. (2004) compared the effects of transplanting allografts of motor, sensory, or mixed nerves to the tibial nerve in rats with a transected tibial nerve. Three weeks after transplantation, nerves receiving motor and mixed nerve grafts showed good histological regeneration, whereas the sensory nerve graft group exhibited poor regeneration, with a significant reduction in the number of nerve fibers, percentage of nerves, and nerve density. This result confirms that there is a significant difference in the regenerative repair capacity of nerve grafts from different tissue sources; however, the exact mechanism for this difference remains unclear. The larger diameter of the intraneural canal in motor nerve grafts facilitates the passage of more regenerated nerve fibers through the nerve defect to the distal end (Moradzadeh et al., 2008). This anatomical advantage may be a key factor contributing to the observed differences in nerve recovery outcomes. Additionally, the regenerative superiority conferred by motor nerve transplants is diminished when the tissue structure of the motor nerve is damaged. This finding suggests that the enhanced regenerative capacity observed with motor nerve grafts may depend on the integrity of the motor nerve’s internal structure (Lloyd et al., 2007).

Axonal regeneration in sensory and motor nerves is governed by their specific phenotypes. When motor and sensory grafts are used separately in peripheral nerve repair, motor grafts preferentially enhance motor axonal regeneration, while sensory grafts improve sensory axonal regeneration. This phenomenon is known as modality-specific regeneration (MSR) (Wood et al., 2015). The concept of modality-specific regeneration emerged from an understanding of preferential motor regeneration, which describes the tendency of motor nerves to reinnervate motor pathways when given equal access to both sensory and motor neural pathways. This phenomenon clearly demonstrates the preference of motor nerves to innervate muscles when provided the opportunity (Madison et al., 2007). In a landmark study, Brushart (1988) showed that motor axons in the rat femoral nerve exhibit a preference for motor pathways following proximal transection. This mechanism is explained by the “pruning hypothesis,” which proposes that regenerating neurons can project multiple axonal collaterals distally. According to this hypothesis, motor nerve collaterals projecting to the motor nerve pathway are preserved, while those projecting to the sensory nerve pathway are pruned, thereby selectively facilitating motor nerve regeneration and the recovery of motor function. However, this selective and beneficial regeneration is only possible in the presence of branches in the damaged nerve; it cannot occur in branchless nerve grafts, as the axon lacks the ability to compare and select between distinct morphologies. Additionally, the trophic effect of the target organ on the nerve, the cellular composition of motor and sensory nerves, and differences in the distribution of trophic factors may account for these differences in regeneration.

The concepts and facts regarding regenerative variations between sensory and motor nerve grafts, as well as pattern-specific regeneration, support the intuition that motor nerves, rather than sensory nerves, should be used for motor nerve repair. However, several studies have reported opposing experimental results, suggesting that the eventual outcome of healing is unaffected by whether the grafts originate from sensory or motor nerves. In a rat model, Kawamura et al. (2010) transplanted sensory, motor, and mixed nerves into pure sensory or motor nerve stumps, respectively, and conducted histological analyses of regeneration in the grafts after 5 to 7 weeks. They were surprised to find no significant differences between these groups. The researchers attributed the discrepancy in findings to the characteristics of the recipient nerves, based on comparisons to earlier trials. In the study by Nichols et al. (2004), the tibial nerve, which is a mixed nerve, received the graft, whereas in Kawamura et al. (2010), a pure sensory or motor nerve was chosen as the recipient. Kawamura et al. (2010) explained that in mixed nerves, sensory and motor nerves compete and interact as they grow in the grafts, leading to better regeneration results for motor nerves, which have larger intimal tubes than sensory nerve grafts. However, when only motor or sensory nerves are present, the different grafts show no significant effect on the outcome due to the lack of interaction or competition.

Another transplantation experiment involving facial nerve injury in rats demonstrated that sensory and motor grafts had a similar ability to promote regeneration of the facial nerve (Ali et al., 2019). In animal experiments, the selection of injured nerves and the observation period following transplantation appear to influence the final conclusions. Whether sensory and motor nerves genuinely affect the recovery process after transplantation requires further research to provide a clearer explanation, thus offering better decision-making criteria for the selection of clinical treatment plans

#### Length of grafts

The length of the graft is critical in determining functional recovery following transplantation. It has been reported that the length of nerve grafts is inversely related to the regenerative capacity of axons (Hoben et al., 2018). In one study, fluorescence imaging of Thy1-GFP rats were used to find out how the length of grafts affect the axonal regeneration (Saheb et al., 2013). It was observed that GFP-labeled axons were able to successfully grow and extend to the distal end of the 20-mm grafts. However, as the length of the grafts increased, a notable decrease in the number of fluorescence-labeled axons reaching the distal part of the grafts was recorded. Specifically, in the 60-mm graft group, most of the fluorescent signals failed to extend beyond the midline of the grafts. This suggests that the growth and extension of axons may be significantly influenced by the length of the grafts, with longer grafts posing greater challenges for axonal growth and signal propagation.

The limited axonal regenerative capacity in long nerve grafts is associated with an increase in senescent cells in the longer nerve grafts (Gordon et al., 2011). These senescent cells, mainly Schwann cells expressing β-galactosidase and p16INK4A, but also macrophages, fibroblasts, and endothelial cells, are widespread in the regions where axonal growth suspends. The mechanism by which increased senescent cells impair nerve regeneration capacity is unclear, however it could be related to long-time denervation of target organs and Schwann cells. In addition, the limited capacity may be related to the rate of revascularization. In animal studies, the use of vascularized nerve grafts showed faster motor unit potential recovery and the appearance of a large number of mature myelinated axons earlier in the regeneration process compared with non-vascularized nerve grafts (Giusti et al., 2016; Giglia et al., 2023).

#### Pre-degeneration treatment of grafts

Following nerve damage, the proximal end initiates a regeneration process, while the distal end undergoes Wallerian degeneration, creating a pro-regenerative microenvironment. Taking advantages of this intrinsic property of nerves following injury, the researchers devised a strategy to significantly enhance the rate of early axonal regeneration in recipient nerves. Specifically, they induced denaturation and a pro-regenerative phenotype in donor nerves by crushing or severing them (Kerns et al., 1993). After allowing the nerves to undergo this transformation process for several days to weeks, the pre-treated nerve fragments were then transplanted into the recipient nerves. This approach effectively promoted early axonal regeneration in the recipient nerves, offering a novel and promising method for accelerating nerve regeneration (Dubuisson et al., 1997). For example, regeneration rates of 1.8–2.1 mm/day have been recorded for pre-degenerate grafts in rats, compared to 1.5 mm/day for fresh nerve grafts (Danielsen et al., 1994). Although pre-degenerate grafts showed functional recovery earlier than conventional autografts, the final functional recovery results were similar to those of conventional autografts. The mechanism by which pre-degeneration promotes axonal regeneration remains unclear, but it could be related to the proliferation of activated Schwann cells and the release of neurotrophic factors in degenerated nerves, both of which reduce the initial delay period and thus accelerate nerve regeneration (Bertelli et al., 2006). In addition, Schwann cells show maximum migratory capacity after 14 days of pre-degeneration (Tomita et al., 2009). All these findings favor the early establishment of axon guidance and improved function after transection injury.

## Decellularized Nerve Grafts Implanted with Cellular Components

Many studies have been conducted on decellularized grafts as a potential alternative to nerve grafts when the defects are too big to repair by autografts (Wei et al., 2022; Matus et al., 2023; Broeren et al., 2024). Since this strategy removes the cellular components of allogeneic or xenogeneic nerve grafts, the immunogenicity of the grafts is eliminated, avoiding the occurrence of severe immune rejection and the use of immunosuppressive drugs. At the same time, it preserves the extracellular matrix, various neurotrophic factors and angiogenic capacity, providing a natural and suitable three-dimensional channel structure for axon regeneration and neural stump connection, which is conducive to the adhesion and growth of regenerated axons to the target organs (Zaminyet al., 2021). The natural structure of decellularized grafts possesses superior biocompatibility, appropriate biodegradability, adequate mechanical strength and elasticity, which is significantly superior to various types of artificial nerve conduit structures (Choi et al., 2018). In conclusion, the use of decellularized grafts for repairing peripheral nerves defects is safe and effective.

There are many methods for peripheral nerve decellularization, each with its own advantages and disadvantages, and there is no widely accepted standard method. The major ways of decellularization include physical methods, such as freezing, freeze-thaw cycle treatment and nonionic detergents, and chemical treatments, such as ionic detergents, amphoteric detergents, and hypertonic and hypotonic solutions. There are many excellent reviews summarizing and comparing in detail the various decellularization techniques currently available, so we will not give a detailed description in this section (Lovatiet al., 2018; El Souryet al., 2021; Susset al., 2022).

**Figure 8:**
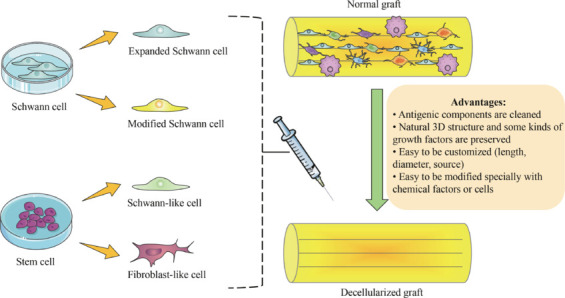
Decellularized nerve and the expanded or modified cells *in vitro.*

Although decellularized grafts are a promising alternative therapy, direct repair of nerve transection injuries with decellularized grafts alone is unlikely to be as effective as autograft repair. Cell therapy has demonstrated remarkable efficacy in peripheral nerve regeneration and repair. Cells that have undergone *in vitro* culture and processing exhibit reduced immunogenicity and can express specific trophic factors that modulate the regenerative environment in a positive manner (Meyer et al., 2008). Researchers have expertly integrated autologous cellular components and decellularized grafts, allowing each component to play an advantage while compensating for their individual shortcomings, significantly enhancing the outcome of nerve graft regeneration (Meng et al., 2024a, b). Currently, the most widely used cellular components include Schwann cells, various stem cells and others, which are described in detail in this section (**Figure 8**).

### Schwann cells

Schwann cells play an irreplaceable and critical role in the regenerative repair process after peripheral nerve injury (Wei et al., 2024). They respond to injury stimuli by undergoing a series of changes, including conversion to a repair phenotype that promotes regeneration, participation in remyelination, and formation of Bungner band, a guiding track for axon regeneration, which directs axon regeneration and accomplishes re-innervation of the target organ.

Schwann cells are a primary biological component and source of immunogenicity in peripheral nerves, and implanting Schwann cells into decellularized nerve grafts has emerged as a highly promising clinically relevant treatment for peripheral nerve injury. When the cultured Schwann cells were put into rat sciatic nerves, they form typical myelin sheaths surrounding axons. Similarly, a 5 mm rat sciatic nerve transection injury was successfully repaired when these cultured Schwann cells were implanted into a semi-permeable catheter (Guénardet al., 1992). Many studies indicate that the combination of Schwann cells and grafts has significant regenerative benefits and pro-repair capabilities, regardless of whether the Schwann cells are derived from autologous, allogeneic, or even xenogeneic sources, whether they are nerve conduits or decellularized grafts, and that *in vitro* culturing appears to reduce the immunogenicity of the Schwann cells and did not cause severe immune rejection in experimental animals (Zhou et al., 2015; Suzuki et al., 2023; Zhang et al., 2024). Although Schwann cells show greater therapeutic potential, further research is needed to determine the best settings for using them to stimulate nerve regeneration. For example, in a previous study, researchers added Schwann cells to a 15-mm decellularized graft but did not see significant nerve regeneration (Fox et al., 2005). The observed negative outcome could be attributed to the relatively small amount of Schwann cells implanted, which was just one-tenth of that used in the study that showed significant improvement. This difference in cell counts could have had a substantial impact on the transplantation procedure’s success, as well as the stimulation of axonal regeneration and functional recovery.

The fact that the axons of motor and sensory nerves exhibit preferential reinnervation properties for their respective target organs suggests that Schwann cells, which play a guiding role in axon regeneration, may be responsible for this difference and may have specific motor and sensory phenotypes. To explore this possibility, Hoke et al. (2006) first transected the cutaneous nerve (a sensory nerve) and the ventral root (a motor nerve), then reinnervated them with cutaneous axons or motor axons. After analyzing each of the three components using reverse transcription polymerase chain reaction for growth factors, they found that nerve growth factor (NGF) and brain-derived neurotrophic factor (BDNF) were predominantly upregulated in cutaneous nerves, whereas pleiotrophin (PTN) and glial cell-derived neurotrophic factor (GDNF) were predominantly upregulated in motor nerves. Based on these differences in the expression of growth factor patterns in Schwann cells, they classified these cells into motor and sensory phenotypes. Furthermore, transplanting sensory axons into the ventral root resulted in a shift in the pattern of growth factor production in the ventral root toward that of sensory nerves, demonstrating the flexibility of Schwann cells under various conditions. A proteomics study revealed that Schwann cells originating from sensory nerves had a more active metabolism and a more developed differentiation phenotype compared to those from motor nerves, despite having lower proliferation potential (Shen et al., 2021). These findings indicate that there are variations between Schwann cells in motor and sensory nerves; however, there are no clear and widely accepted indicators or guidelines to distinguish these two types of Schwann cells (Hercher et al., 2019). Nevertheless, strong phenotypic commitment has been reported for motor and sensory Schwann cells *in vitro* (Hercher et al., 2020). This ability to retain their original phenotype aids in axon-specific regeneration, particularly when the phenotype of the Schwann cells matches that of the grafted nerves, allowing for faster reinnervation of the target organ. Given these contentious findings, more research is needed to determine whether the motor or sensory phenotype of Schwann cells can be altered by local microenvironmental effects. Additionally, the differences and specific markers of motor and sensory nerve-derived Schwann cells should be further investigated.

Based on previous research into the motor and sensory phenotypes of Schwann cells, Schwann cells from various sources were transplanted into cryopreserved decellularized nerve grafts following *in vitro* culture to determine the impact of different Schwann cells on nerve repair (Jesuraj et al., 2014). Approximately 6 weeks after transplantation, the regenerating nerves from both phenotypic Schwann cell groups exhibited equal regenerating fiber density and axon diameters, although these dimensions were slightly smaller than those of allogeneic nerve grafts. At 12 weeks post-transplantation, both groups showed a significant increase in muscle strength recovery, reaching levels comparable to allogeneic grafts. These findings suggest that Schwann cells from various sources have a similar beneficial impact on peripheral nerve regeneration. The susceptibility of Schwann cells to adaptive remodeling by environmental influences during the re-differentiation process, along with their gradual change in gene expression patterns during *in vitro* proliferation, are important factors that determine the ultimate fate of Schwann cells. Given the potential impact of these parameters, it appears that primary Schwann cells that have not undergone *in vitro* proliferation are the best choice for studying the effects of motor or sensory-derived Schwann cells on nerve regeneration.

As we know, rodents possess a strong ability for neurological regenerative repair; consequently, nerve transplantation investigations in rodents often produce better outcomes than clinically predicted (Angius et al., 2012). Decellularized allogeneic nerve grafts implanted with Schwann cells significantly improved the functional and histological recovery of the rat sciatic nerve, yielding results comparable to autografts (Aszmann et al., 2008). To further study whether Schwann cells combined with decellularized grafts exhibit excellent pro-regenerative effects in larger mammals, various transplantation trials have been conducted in non-human primates.

Hess et al. (2007) investigated this using ten distantly interbred Cynomolgus monkeys. They created a 6-cm-long ulnar nerve defect model and decellularized the grafts by freezing them for seven weeks. Depending on the treatment of the grafts, the animals were divided into three groups: decellularized grafts inoculated with Schwann cells, grafts without Schwann cells, and autologous fresh grafts. The types and amounts of cytokines detected were found to be approximately equivalent across all three experimental groups. Six months after neural continuity reconstruction, the number, density, and percentage of nerve fibers were notably higher in grafts inoculated with Schwann cells compared to those that were not. Nevertheless, both of these groups still fell short of the recovery results achieved with autologous fresh grafts. Levi et al. (2016) described, for the first time, the use of a complex nerve transplant composed of autologous cultivated Schwann cells and peroneal nerve grafts to repair a severe sciatic nerve deficit of up to 7.5 cm in a clinical patient. During a 2-year postoperative follow-up, the continuity of the nerve graft repair and the absence of neural tumor formation were assessed using ultrasound and magnetic resonance imaging. Furthermore, the patient showed evidence of proximal sensory restoration and definitive distal motor restoration in the tibial and common peroneal nerve distributions, indicating that the transplantation of autologous Schwann cells has therapeutic safety and practicality. Although autologous Schwann cells combined with decellularized nerve grafts have not yet demonstrated the same recovery as autologous nerve grafts in non-human primate and human clinical studies, these positive signs of regeneration continue to encourage further development of this therapy as a clinically promising alternative to autologous nerve grafts for the treatment of larger nerve deficits (Hess et al., 2007).

To achieve more effective nerve repair, certain researchers have conducted cellular engineering modifications on the transplanted Schwann cells. These modifications enable the high expression of genes associated with nerve regeneration within the Schwann cells. As a result, the nerve regeneration process is accelerated, and the prognosis is significantly improved. In a particular study, purified adult rat Schwann cells underwent transfection with genetically modified viral vectors (Godinho et al., 2013). This process resulted in the expression of distinct neurotrophic factors within the Schwann cells: BDNF, ciliary neurotrophic factor (CNTF), and neurotrophic factor NT3. Subsequently, these genetically modified Schwann cells were combined with decellularized grafts to repair a 1-cm gap in the unilateral peroneal nerve of adult rats. The different neurotrophic factors exhibited varying effects on nerve morphology, the number of regenerating axons, myelin formation, and motor function. For instance, NT3 grafts demonstrated the highest number of sensory fibers, while CNTF grafts exhibited the fewest axons. This study provides some fascinating inspiration. First, the Schwann cell is one of the most important cells in the regeneration and repair of peripheral nerves, and several researches have been conducted on the function of Schwann cells and their related trophic factors. The properly application of these existing high-quality discoveries to achieve specific nerve graft repair by gene editing or alteration appears exceedingly promising. Second, reprogramming of Schwann cells occurs during injury and regeneration, and the local environment influences the gene expression pattern of Schwann cells, which can be controlled by epigenetic regulation. Furthermore, with the advancement of tissue engineering techniques and cellular molecular research tools, researchers may clarify the precise gene expression of Schwann cells in a specific disease or state, allowing for targeted artificial modifications (Wang et al., 2017). For example, Li et al. (2022) identified by RNA sequencing the highly differentially expressed transcription factor POU6F1 that appears in nerves after repair of decellularized nerve grafts. Subsequently, they used adeno-associated virus to transfect Schwann cells to make them highly express POU6F1. When modified Schwann cells were combined with decellularized grafts for the repair of sciatic nerve lesions, axonal regeneration, myelin production, and functional motor recovery were all improved compared to the combination of unmodified Schwann cells and acellular nerve allograft. Gene editing is an innovative technology of great value and significance, and a number of clinical studies have reported important breakthroughs and achievements in transplanting visceral organs from animals that have undergone multi-gene editing into clinical patients (Sykes et al., 2022; Harisa et al., 2023; Wang et al., 2024). Peripheral nerve grafts may be able to achieve efficient and personalized repair through gene editing in the future as well.

### Mesenchymal stem cells

Theoretically, autologous Schwann cells are the most suitable transplantation cells for nerve repair. Although much has been accomplished in this area, there are still many challenges in the operation from the harvesting of the Schwann cells to the final transplantation. Severely damaged peripheral nerves often require trimming of the nerve stump prior to surgical closure to remove structurally disorganized portions of the nerve to obtain a good alignment (Anderson et al., 2017). Autologous Schwann cells are usually harvested during this process to avoid additional damage to the normal nerve. However, the purity and quantity of Schwann cells acquired via this approach are low. To reach the requisite number of cells, *in vitro* proliferation is required (Rodríguez et al., 2000). Extending the expansion process and *in vitro* culture period can have a considerable impact on the differentiation status and activity of Schwann cells, as well as altering their gene expression patterns. Furthermore, protocols for isolating and purifying Schwann cells have not been standardized, making it challenging to obtain cells that match the requirements in a consistent and repeatable way.

Given the numerous challenges associated with the use of Schwann cells, the search for alternative cell types that can replace them, or the artificial induction of other cell types to develop a Schwann cell-like phenotype, appears to be a highly promising and viable strategy.

The ideal “transplantable cells” are expected to possess the following characteristics: readily accessible, capable of rapid proliferation in culture, immunologically inert, capable of long-term survival and integration within host tissues, and even capable of stable transfection and expression of exogenous genes (Millesiet al., 2007; Li et al., 2021c). Mesenchymal stem cells (MSCs) have attracted much attention as the most promising replacement cells due to their great growth and differentiation potential, easy accessibility and ability to be induced into neural support cells (Ding et al., 2011). MSCs can be obtained from many parts of the body and have been isolated from bone marrow, adipose tissue, peripheral blood, amniotic fluid, umbilical cord, tendons and ligaments, hair follicles, synovial membranes, olfactory mucous membranes, dental pulp and fetal tissues (Hass et al., 2011; Via et al., 2012). MSCs are innately capable of differentiating into all mesodermal lineages: fat, bone, muscle and cartilage. Under appropriate circumstances, MSCs differentiation can be directed to non-mesenchymal lineages such as neurons, astrocytes, and Schwann-like cells that produce and secrete neurotrophic factors and build extracellular matrix components to support nerve regeneration (Scuteri et al., 2011). The artificial induction of MSCs into Schwann cell-like cells necessitates a pre-differentiation treatment. However, this approach has notable drawbacks, including the requirement for additional preparation time and increased costs, which collectively render the method less amenable to clinical applications. Moreover, a substantial amount of research is still required to elucidate several critical factors prior to the initiation of clinical studies involving stem cell transplantation (Poulos et al., 2018; Chu et al., 2020; Zhou et al., 2021). These factors include the optimal dosage, the most effective method of administration, the *in vivo* survival rate of the transplanted cells, and the underlying mechanisms through which these cells interact with the host environment (Jiang et al., 2017). Currently, bone marrow mesenchymal stem cells (BM-MSCs) and adipose-derived stem cells have been relatively well-studied and will be the focus of this section. Other MSCs will not be discussed in detail in this section and can be found in some excellent comprehensive reviews (Bojanicet al., 2020; Yi et al., 2020; Jiang et al., 2022).

#### Bone marrow mesenchymal stem cells

In a study involving the repair of a 40-mm defect in the ulnar nerve of the non-human primate rhesus monkey, researchers achieved significant structural and functional improvements by implanting cultured autologous BM-MSCs into decellularized nerve grafts (Zhao et al., 2011). Notably, these improvements were observed six months post-transplantation. Measurements of peak compound muscle action potential (CMAP) amplitude, CMAP latency, and nerve conduction velocity revealed no significant differences between the group repaired with decellularized allogeneic nerve grafts implanted with autologous BM-MSCs or Schwann cells and the group repaired with autologous nerve grafts. In other studies, particularly those using smaller animal models, it was found that the combination of BM-MSCs with catheters or nerve grafts led to increased axonal regeneration and improvements in wet muscle weight and walking track scores, achieving results comparable to autografts (Ye et al., 2022, 2025; Liang et al., 2023). The combination of BM-MSCs with decellularized grafts has demonstrated superior recovery outcomes in both rodent and non-human primate models (Li et al., 2016b). This consistent efficacy across species suggests broad application potential and translational promise, particularly in repairing larger nerve lesions.

BM-MSCs can differentiate into a Schwann cell-like phenotype that helps promote nerve repair and this process is modulated by the physiological microenvironment at the transplantation site. BrdU-labeled undifferentiated BM-MSCs were transplanted into the distal stump of the transected sciatic nerve by fiber injection, and close to 5% of BrdU-positive cells exhibited a Schwann cell phenotype when tested 33 days after transplantation (Cuevas et al., 2002). Although these results demonstrate the ability of BM-MSCs to actively differentiate into Schwann cells and contribute significantly to the regenerative repair of the nerve, only a minority of the cells were differentiated and mature. In contrast, artificial induction of differentiation of BM-MSCs *in vitro* is a more efficient and feasible option (Hou et al., 2006). The *in vitro* pre-differentiation of BM-MSCs towards a Schwann cell-like phenotype, achieved either through chemical induction or by some specific growth factors, has been demonstrated to be a more efficient approach to augment the population of Schwann cells (Lin et al., 2008). Dezawa et al. (2001) reported the first successful *in vitro* induced differentiation of bone marrow BM-MSCs into cells exhibiting a Schwann cell-like phenotype and capable of forming myelin. This groundbreaking study further demonstrated that these differentiated cells could effectively promote peripheral nervous system regeneration and myelin re-formation in a rat model of sciatic nerve transection injury. Schwann-like cells induced from BM-MSCs have been shown to contribute more significantly to nerve regeneration compared with BM-MSCs themselves (Fan et al., 2014). Furthermore, when these induced BM-MSCs are combined with autologous nerve grafts, the final recovery of injured nerves is nearly equivalent to that achieved with autografts alone. These results support the feasibility and efficacy of converting mesenchymal stem cells into Schwann cell-like cells. However, it is still uncertain whether these induced stem cells stimulate nerve repair and regeneration via the same mechanisms as Schwann cells.

Although a variety of stem cells can be induced to differentiate into Schwann cells-like cellular phenotypes *in vitro* and exert positive effects on nerve repair, these cells do not seem to be able to truly replace the functions of Schwann cells. Recent studies have indicated that in the complex *in vivo* environment, Schwann cells differentiated from BM-MSCs exhibit significant differences compared to autologous Schwann cells in terms of their contributions to nerve regeneration and axonal myelination following nerve transection injury (Ladak et al., 2011; Hou et al., 2018b). When decellularized grafts were inoculated with autologous Schwann cells rather than Schwann cell-like cells differentiated from BM-MSCs, rats exhibited significantly higher levels of myelin thickness, myelinated fiber ratio, and myelin area ratio in the regenerating nerves. This result suggests that Schwann cells differentiated from BM-MSCs cannot completely replace autologous Schwann cells, and that there are differences in the mechanisms and effects of the two in promoting nerve regeneration.

In addition to differentiating into Schwann cell–like cells, BM-MSCs can also differentiate into fibroblast-like cells, which are major components of peripheral nerves (Chen et al., 2007). These fibroblast-like cells can synthesize and secrete a variety of trophic factors and supportive substances after implantation into the sciatic nerve, including nerve growth factor, brain-derived neurotrophic factor, glial cell line-derived neurotrophic factor, ciliary neurotrophic factor, collagen, fibronectin, and laminin, all of which have been shown to promote nerve regenerative repair. Furthermore, administering BM-MSCs to nerves also promotes angiogenesis (Han et al., 2016). Due to their strong differentiation potential, MSCs can develop into multiple cellular components in peripheral nerves, thereby participating in the nerve repair process and demonstrating superior clinical translational prospects.

The development of decellularized nerve grafts has significantly alleviated the issue of limited donor nerve availability in autologous nerve transplantation. Decellularized grafts, which lack cellular antigenic components, can theoretically be derived from any species after decellularization. Additionally, these grafts are capable of accommodating nerve repair across a broad range of sizes. Some researchers have investigated the combination of BM-MSCs and xenogeneic acellular nerve grafts to determine whether they can replace the use of autografts (Hou et al., 2018a). The functional recovery of nerves in the autograft group was significantly superior to that of the xenogeneic acellular grafts implanted with BM-MSCs, as measured by the sciatic nerve function index and electrophysiologic data. However, when the histomorphology of regenerated nerves was evaluated, both the autograft group and the xenograft implanted with BM-MSCs group showed well-defined myelin re-formation, as well as increased axon quantity and myelin thickness, outperforming the xenograft group. This result is promising, as the histological data indicated that BM-MSCs could promote remyelination similar to Schwann cells and facilitate axonal regeneration, regardless of the graft source, although functional recovery did not meet expectations. Interestingly, improved histologic morphology does not guarantee functional recovery of peripheral nerves, and several other research findings support this conclusion (Deumens et al., 2010; Brogan et al., 2021). Although the restoration of tissue structure is the basis for functional recovery, it does not determine the final functional outcome. This phenomenon poses a complex dilemma for the repair and regeneration of peripheral nerves, and the underlying reasons and mechanisms should be investigated more thoroughly.

Although BM-MSCs possess substantial potential and are currently one of the most extensively studied stem cell types in the field of neural regeneration, their application is constrained by the invasive nature of autologous collection procedures (Nellinger et al., 2023; Boysen et al., 2024). The acquisition process is invasive and often painful, typically necessitating anesthesia. Moreover, the yield of BM-MSCs obtained from this source is significantly lower compared to other sources. While BM-MSCs are more easily obtained than embryonic and neural stem cells and have fewer ethical concerns, they exhibit lower proliferation capacity and differentiation potential. Additionally, stem cells are associated with the risk of uncontrolled proliferation, which may potentially lead to tumorigenesis.

#### Adipose-derived mesenchymal stem cells

Adipose-derived mesenchymal stem cells (AD-MSCs) can be easily obtained from abundant adipose tissue through minimally invasive methods such as liposuction. They exhibit better stem cell ratios, proliferation, and differentiation potential compared to bone marrow-derived mesenchymal stem cells (BM-MSCs) (Bunnell et al., 2021; Gopalarethinam et al., 2023). AD-MSCs are pluripotent like BM-MSCs and can be induced to differentiate into a Schwann cell-like phenotype (Sumarwoto et al., 2023). These differentiated AD-MSCs display functional and morphological characteristics comparable to those of autologous Schwann cells and possess a similar level of potency. When combining denervated nerve grafts with AD-MSCs or BM-MSCs, both grafts exhibited the same potential to stimulate nerve regeneration and repair. Although they did not reach the pro-regenerative level of autologous grafts, both surpassed the repair results of the denervated graft group alone (Zhou et al., 2020). It is hypothesized that AD-MSCs promote the recruitment of endogenous Schwann cells by expressing various growth factors, such as nerve growth factor, vascular endothelial growth factor, and brain-derived neurotrophic factor. This mechanism is believed to result in long-term therapeutic effects that facilitate neural regeneration and protection beyond the lifespan of the AD-MSCs themselves. AD-MSCs are currently regarded as one of the most practical sources for obtaining stem cells and have demonstrated significant translational potential for clinical applications, a trend expected to continue. In addition to their practicality, these cells exhibit low immunogenicity and can maintain long-term plasticity and phenotypic stability *in vitro* (Naderi et al., 2017). Another important advantage is that the anatomical site from which the cells are harvested and the age of the donor do not significantly influence the therapeutic outcome (Li et al., 2021a). For these reasons, AD-MSCs are attractive for regenerative medicine, tissue engineering, and peripheral nerve regeneration. MSCs can be induced to differentiate into Schwann cell–like cells to promote the regenerative repair of peripheral nerves. However, the *in vitro* induced differentiation process for stem cells is time-consuming and costly, requiring weeks of processing to meet the requirements for use. During this period, target organs and Schwann cells remain denervated, which severely affects the prognosis and functional recovery of nerve repair and misses the optimal time for surgical intervention and treatment. To optimize the application of stem cells for peripheral nerve regeneration, researchers have investigated both differentiated and undifferentiated AD-MSCs to ascertain whether there are disparities in their capacity to promote peripheral nerve regeneration. Mathot et al. (2021) extracted and purified MSCs from rat inguinal fat pads, triggering differentiation of one set of MSCs into Schwann cell-like cells while the other group remained undifferentiated and primitive. By combining with decellularized nerve grafts, a 1-cm sciatic nerve defect was repaired in rats. At 12 weeks, undifferentiated MSCs significantly improved isometric tonic force measures and compound muscle action potential outcomes compared to decellularized allogeneic grafts alone, whereas differentiated MSCs greatly enhanced compound muscle action potential. At week 16, function restored to normal in all groups. At both time points, the pro-neural regeneration effects of undifferentiated versus differentiated MSCs were not statistically different, with outcomes similar to autografts in most assessments.

### Induced pluripotent stem cells

During the past 20 years, the development of stem cell technology has expanded the cell sources for cell therapy, greatly advancing the progress of regenerative medicine. In particular, Takahashi et al. (2006) achieved a landmark breakthrough by inducing pluripotent stem cells (iPSCs) from adult somatic cells, laying the foundation for studying the molecular mechanisms and signaling pathways of human cells *in vitro*. The induction of somatic cells has enabled scientists to avoid immune and ethical issues, allowing them to obtain large quantities of cells non-invasively.

In the field of peripheral nerve regeneration and repair, various methods have been reported to induce induced pluripotent stem cells (iPSCs) to differentiate into cell types that promote nerve regeneration (Huang et al., 2020; Powell et al., 2021; Yokoi et al., 2021). These methods can produce high-purity, large quantities of target cells, providing a methodological foundation for the development of cell therapy in peripheral nerve repair. These cell types include neural crest stem cells, Schwann cells, mesenchymal stem cells, and sensory neurons (Sayad-Fathi et al., 2019; Yi et al., 2020; Marshall et al., 2024; Pan et al., 2025). Liu et al. (2012a) reported the induction and expansion of nearly pure Schwann cell populations from iPSCs. These Schwann cells can be cryopreserved and promote myelin formation in primary neurons during co-culture. However, the function of Schwann cells differentiated from neural crest stem cells (NCSCs) remains limited, with only a small proportion of NCSC-derived Schwann cells producing functional myelin-forming cells, necessitating further optimization and refinement of the induction and differentiation steps. Additionally, Kim et al. (2017) developed a method to generate myelin-forming Schwann cells from iPSCs. After co-culturing with rat dorsal root ganglia neurons, the iPSC-derived Schwann cells tightly bound to axons, suggesting myelin formation *in vitro*. By transplanting induced Schwann cells into mice with sciatic nerve injuries, nerve fiber regeneration is promoted, and the transplanted cells integrate into the regenerated axons, enhancing the therapeutic effects of peripheral nerve regeneration.

Over the past decade, there have been reports of using the inducible differentiation properties of iPSCs in combination with nerve scaffolds such as decellularized grafts or artificial conduits to repair severed nerves, with promising results (Yokoi et al., 2018; Mitsuzawaet al., 2020). iPSCs can be induced to differentiate into neural crest stem cells, which can further differentiate into Schwann cells (Wang et al., 2011). Kimura et al. (2018) combined iPSC-derived Schwann cells with artificial nerve conduits and observed significant promotion of axonal regeneration and remyelination in a mouse model of 6 mm long nerve defects, while also increasing vascularization within the conduits. The experimental group achieved similar motor function recovery outcomes to the autologous nerve transplantation group, suggesting that iPSC-derived Schwann cells represent a promising approach for treating large peripheral nerve defects. Huang et al. (2017) further investigated the different stages of differentiation from iPSCs to neural crest stem cells and then to Schwann cells, exploring the respective effects of neural crest stem cells and Schwann cells derived from iPSCs on peripheral nerve regeneration and repair. These cells were respectively combined with fiber conduit scaffolds and used to repair a 10 mm long nerve defect in rats. Interestingly, the results showed that neural crest stem cells derived from iPSCs were more effective than Schwann cells in promoting early functional recovery of motor nerves, and yielded better long-term muscle recovery outcomes. This finding appears to be related to the differentiation and paracrine signaling of neural crest stem cells. In complex regeneration and repair environments, allowing cells with differentiation potential to autonomously adapt to local conditions may be more beneficial for ultimate functional recovery than artificially inducing directed differentiation. The mechanisms underlying the differing outcomes of functional recovery across various stages of iPSC-induced differentiation require further clarification in the future, thereby establishing more precise application standards for the use and development of cell therapy in clinical settings. Based on the results of the combined use of iPSCs and nanofibrous tubes discussed above, it can be speculated that decellularized nerve grafts with natural nerve structures and biomechanical properties may achieve better regeneration and functional recovery outcomes, which may be validated in the future.

iPSCs can also be induced to differentiate into bone marrow mesenchymal stem cells, which subsequently promote peripheral nerve regeneration by facilitating angiogenesis in decellularized grafts. Meng et al. (2024a, b) used a combination of iPSC-derived mesenchymal stem cells (iMSCs) and decellularized nerve grafts to promote the recovery and regeneration of a 10 mm defect in the rat sciatic nerve. The motor recovery results of the iMSCs and decellularized grafts combination group were comparable to those of the autologous graft group. Since certain neurotrophic factors such as BDNF, NGF, and vascular endothelial growth factor (VEGF) were significantly increased in the combination group, Meng et al. (2024a, b) proposed that the neurotrophic factors secreted by iMSCs in response to the repair process in this environment are key to accelerating axonal regeneration and angiogenesis in decellularized grafts. However, the specific molecular mechanisms remain to be further elucidated.

Although a long-term follow-up study showed that iPSCs can enhance axonal regeneration and myelin formation at 24 and 48 weeks post-surgery without inducing teratomas, iPSCs still exhibit malignant potential similar to that of embryonic stem cells (Okita et al., 2007; Nakagawa et al., 2008; Uemura et al., 2014). Furthermore, the methods and techniques for producing induced pluripotent stem cells that meet demand still need to be further optimized. (Louitet al., 2023).

### Neural stem/progenitor cells

In fact, a key feature of stem cells used for peripheral nerve injury repair is their ability to differentiate into Schwann cell-like cells, thereby promoting axonal regeneration and remyelination, or promoting nerve regeneration by regulating Schwann cell activity and secreting neurotrophic factors.

Neural stem cells (NSCs) are an important component of the central nervous system and have two fundamental characteristics: self-renewal and pluripotency (Molofskyet al., 2003). During development, NSCs differentiate into radial glial cells and proliferate into neural progenitor cells (NPCs) (Kawaguchi et al., 2020). Neural stem cells and progenitor cells (NSPCs) have been identified as the only self-renewing cell types in the adult central nervous system. NSCs can not only differentiate into neurons, astrocytes, and oligodendrocytes but also into Schwann cell-like cells, promoting the outward growth of injured peripheral nerve axons (Liu et al., 2023a). NSPCs can also secrete various neurotrophic factors, including BDNF, fibroblast growth factor (FGF), NGF, insulin-like growth factor (IGF), and hepatocyte growth factor (HGF) (Finkel et al., 2021; Jeon et al., 2024). Additionally, NSPCs promote axonal myelin regeneration, angiogenesis, and immune regulation (Miller et al., 2025). Therefore, it is theoretically possible that NSPCs can promote the repair of peripheral nerve injury through multiple pathways. Here, we will discuss NSCs and NPCs together.

NSCs were first isolated from the brains of adult mice, and have since been successfully isolated from humans and non-primate animals (Dumitru et al., 2025). NSCs have demonstrated their potential for application in the treatment of peripheral nerve injury (Liard et al., 2012; Ni et al., 2013). Murakami et al. (2003) obtained nestin antibody-positive neuroblasts from cultured fetal mouse hippocampal neural progenitor cells. These cells not only differentiate into GFAP-positive astrocytes, galactocerebrosidase-positive oligodendrocytes, and neurofilament 200-positive neurons, but also into Schwann-like supporting cells positive for anti-s100 and anti-p75 antibodies. The obtained NPCs were embedded in silicone tubes and used to treat a 15 mm long sciatic nerve defect in rats. At 6 and 10 weeks post-transplantation, the number and diameter of myelinated fibers were significantly higher in the combination group than in the cell-free catheter group, and action potentials were successfully detected in the regenerated nerves. These results confirm that NPCs derived from fetal rat hippocampus retain their proliferative and differentiative capabilities in collagen scaffolds. When transplanted into peripheral nerve defects, a portion of NPCs differentiate into Schwann cell-like cells, thereby promoting axonal regeneration. Lee et al. (2017) combined artificial nerve conduits with NSCs to repair a 5 mm defect in the sciatic nerve of mice. They found a 1.6 times higher expression level of IL-12p80 in the combination group than in cell-free nerve conduits group. The combination of neural tubes and NSCs improved motor function recovery in mice and increased the diameter of regenerated nerves after sciatic nerve injury. Lee et al. (2017) further demonstrated that IL-12p80 can induce NSCs to differentiate into Schwann cells through Stat 3 phosphorylation, thereby confirming the potential of IL-12p80 in the clinical treatment of sciatic nerve injury.

One of the challenges limiting the widespread use of NSPCs is the difficulty of harvesting them directly from living organisms. Inducing differentiation of stem cells such as MSCs and iPSCs into NSPCs has become an important source for obtaining sufficient NSPCs (Bell et al., 2019; Hörneret al., 2021; Koh et al., 2024). For example, Zhang et al. (2017) directly induced NPCs from human gingival-derived mesenchymal stem cells. After transplanting gingival-derived mesenchymal stem cells and NPCs into crush-injured sciatic nerve respectively, the gingival-derived mesenchymal stem cells differentiated into neuron cells, while the NPCs differentiated into both neurons and Schwann cells. NPCs demonstrated superior efficacy in promoting regeneration at the injury site and in the distal axons of the injured sciatic nerve compared to gingival mesenchymal stem cells. Onodeet al. (2021) were the first to combine biodegradable nerve conduits with iPSC-derived neurospheres containing NSPCs, demonstrating their regenerative efficacy in the 5 mm long sciatic nerve defect in rats. The iPSC-derived neurospheres persisted in the graft for over 8 weeks and completely disappeared by week 12. At 12 weeks, there was no significant differences in sensory nerve regeneration between the combination and autograft groups. As for motor function recovery, the combination group was significantly superior to that in the cell-free conduit group, though it did not reach the same level as the autograft group. Histologically, the combination group exhibited a greater number and size of axons compared with the cell-free conduit group. These findings confirm that iPSCs-derived NSPCs combined with the neural conduit hold promise for regenerative repair applications; however, the specific mechanisms underlying this regenerative effect require further clarification. In another study, researchers used iPSC-derived NPCs to treat optic nerve glial cell injury in an optic nerve crush model (Park et al., 2021). They found that NPCs exhibit significant neuroprotective and regenerative effects both *in vivo* and *in vitro*. Compared with iPSCs, NPCs exhibit superior neuroprotective and regenerative effects, which are associated with NPCs inducing greater expression of Iba1 in retinal microglia and their migration into the retina. These activated microglia participate in axonal regeneration and clearance processes within the retina.

In addition, some studies have reported that overexpressing different combinations of neural lineage-specific transcription factors can reprogram fibroblasts into neural progenitor-like cells (Han et al., 2012; Lujan et al., 2012; Tan et al., 2025). Although they observed beneficial effects on peripheral nerve regeneration, they still hold the tumorigenic risk associated with the exogenous introduction or overexpression of related functional genes. One issue limiting the clinical application of NSPCs is the high risk of neuroblastoma (Deng et al., 2018). Johnson et al. (2008) reported that nearly a quarter of subjects developed neuroblastoma when immortalized mouse neural stem cells were used to treat sciatic nerve defects and crush injuries, a phenomenon that may be related to the overexpression of growth factors.

In conclusion, NPSCs can promote peripheral nerve regeneration by differentiating into Schwann cell-like cells, secreting nutrients, and promoting angiogenesis, while the underlying specific molecular mechanisms remain unclear. Optimizing the induction and reprogramming steps to increase the *in vivo* stability of NPSCs and reduce their tumorigenicity is key to expanding their application prospects.

### Factors affecting the prognosis of decellularized nerve grafts

Decellularized nerve grafts are an effective alternative to nerve grafts and can overcome some of the dilemmas faced by autologous or allogeneic grafts. The most important characteristic of decellularized nerve grafts is the elimination of cellular components that possess immunogenic properties, while retaining the native scaffolding structure and a subset of chemokines within the extracellular matrix. This preservation provides essential structural support for axonal regeneration. Nevertheless, the neuroregenerative efficacy of decellularized nerve grafts is influenced by a multitude of factors. These include the specific decellularization methodology employed, the length of the decellularized grafts, the health status of the graft donor, the recipient’s lifestyle habits, as well as the type and anatomical location of the nerve receiving the graft. Here, we will only introduce the first two main factors, and the other factors can be found easily in a number of excellent reviews (Carré et al., 2022; Zhu et al., 2023).

#### Decellularization strategy

During the fabrication of acellular neural matrix grafts, physical, chemical, and enzymatic treatments can exert substantial effects on the biological properties of the nerve tissue (Zhang et al., 2021b; Mahdian et al., 2023). Here, we are going to discuss about these influences (**Additional Table 2**).

**Additional Table 2:** Summary of major methods for decellularization of peripheral nerves

Method		Mechanism	Effect on ECM	Advantage	Disadvantage
Physical	Cold preserve (Fox et al., 2007)	Reduction of immunogenicity by progressive breakdown of cells	ECM can be disrupted or fractured during rapid freezing.	Simple operating process	(1) Freezing temperatures and times need to be explored. (2) Preparation time is usually 21 d and above. (3) Cells are not killed. (4) Cells are not cleared from the ECM and may trigger an immune response.
	Freeze-thaw (Krekoski et al., 2001)	Disruption of cell membrane due to the formation of intracellular ice crystals	ECM can be disrupted or fractured during rapid freezing.	(1) Simple operating process (2) Fast, usually around 10 min	(1) Cells are not cleared from the ECM and may trigger an immune response. (2) ECM structure is heavily damaged by ice crystals.
	Supercritical carbon dioxide (Choi et al., 2024)	Supercritical fluids have unique transport properties of liquid-like density and gas-like diffusivity.	Maintains ECM proteins and mechanical properties	(1) Fast, within 1 h (2) Well preserved composition and structure of ECM	Higher operational and equipment requirements
Chemical	Non-ionic detergents (Sondell et al., 1998)	Disruption of lipid-lipid and lipid-protein interactions	Triton X-100: ECM would be disrupted with a loss of laminin and glycosaminoglycans	Sondell method: Cells are removed more efficiently.	Sondell method: (1) Complicated operation process (2) ECM is destroyed. (3) Chemical residue
	Ionic detergent (Hudson et al., 2004)	Solubilization of cell membranes and disruption of protein-protein interactions	(1) Sodium dodecyl sulfate (SDS): Removes nuclear remnants and cytoplasmic proteins; tends to disrupt native tissue structure, remove glycosaminoglycans and damage collagen Sodium deoxycholate: More disruptive to tissue structure than SDS Triton-200X: Efficient cell removal with ECM disruption similar to that of Triton X-100	Hudson method: Well preserved composition and structure of ECM	Hudson method: (1) The process is complex. (2) Incomplete cell clearance (3) Triton-200X discontinued chemical residue.
	Zwitterionic detergents (Hudson et al., 2004)	Display properties of both ionic and non-ionic detergents	Sulfobetaine-10 and-16 (SB-10, SB- 16): Yielded cell removal and mild ECM disruption with Triton X-200		
Enzymatic	Nucleases (Bae et al., 2021)	Catalyze the hydrolysis of the interior or terminal bonds of ribonucleotide and deoxyribonucleotide chains	Difficult to remove from the tissue and could invoke an immune response	Facilitate removal of nuclear material and increases decellularization efficiency	Need to be used in conjunction with physical or chemical methods
	Chondroitinase ABC (Krekoski et al., 2001)	Removal of chondroitin sulfate glycosaminoglycans	It can remove chondroitin sulfate glycosaminoglycans in ECM	Significantly increase length of axon growth in decellularized nerve grafts	Need to be used in conjunction with physical or chemical methods

ECM: Extracellular matrix; SDS: sodium dodecyl sulfate.

Physical decellularization methods include pre-denaturation, cold preserve, freeze-thaw. The immunogenicity of cold-preserved allografts has been demonstrated to diminish with extended periods of freezing, which has been extensively investigated across a range of animal studies. While both the temperature and duration of freezing are critical factors affecting the resulting immunogenicity, temperature appears to have a more significant influence (Krekoski et al., 2001; Fox et al., 2007). These physical methods can only kill or reduce the immunogenicity of the cells, but cannot completely remove the cells from the nerve graft. Therefore, when the grafts are likely to remain partially immunogenic after treatment, the use of immunosuppressive agents is required. Damaged cells and debris persist within the extracellular matrix (ECM) of neural tissue, failing to be effectively cleared. Upon implantation of a graft, these residual fragments may trigger the activation and infiltration of Schwann cells, macrophages, and other inflammatory cells into the basement membrane tube. This inflammatory response has the potential to degrade the graft material, induce damage to the basement membrane tube, and impede the normal regenerative process of axons.

Supercritical carbon dioxide (scCO_2_) possesses unique transport properties, characterized by its liquid-like density and gas-like diffusivity. These properties enable rapid solvent release, preventing retention within the tissue and thereby eliminating the necessity for extensive washing procedures (Massias et al., 2023). Given that CO_2_ is non-polar, ethanol can be introduced as a co-solvent to facilitate the removal of polar phospholipid components from cell membranes. Following treatment, both nuclei and cell membranes were undetectable in the tissues. Moreover, the content of collagen and elastin, as well as the mechanical strength of the tissues, remained unchanged. These findings demonstrate that scCO_2_ may exert minimal damage to the ECM. In an effort to address the limitations of conventional decellularization techniques, Choi et al. (2024) used scCO_2_ to reduce both the processing time and the risk of chemical residue retention. The efficacy of scCO_2_-based decellularization was subsequently compared with that of Hudson’s decellularization method. The expression levels of double-stranded DNA (dsDNA), major histocompatibility complex (MCH)-I (MHC I), and (MHC II) were significantly diminished in the decellularized nerves following treatment, confirming the effective removal of cellular debris. Additionally, the preservation of extracellular matrix proteins and various factors was superior in the scCO_2_ group compared with the detergent group. Moreover, nerve regeneration was enhanced in the scCO_2_ group. This improvement may be attributed to the higher retention of various chemokines in the scCO_2_ group, including angiopoietin-1, vascular endothelial growth factor and granulocyte-macrophage colony-stimulating factor.

Chemical decellularization is a detergent-based technique that effectively eliminates cellular and myelin components from nerve tissue. This process essentially removes antigenicity while preserving the basement membrane and lamellar structure of the nerve. As a result, the decellularized nerve serves as an optimal scaffold for nerve regeneration. The widely known Sondell method (Sondell et al., 1998), for example, uses a process that includes Triton X-100 and deoxycholate to remove cellular components. This decellularized graft supports axonal growth and migration of Schwann cells, which reoccupy empty basement membrane tube without signs of excessive inflammation. However, the chemicals usually cause some degree of damage to the structure of the nerve basement membrane and ECM, and therefore may affect axonal regeneration. In Sondell method, there was marked damage to the gross morphology and basement membrane of the grafted nerves. The grafted nerve tissue was markedly crumpled in deoxycholate and swollen in distilled water, which may have contributed to the structural damage. On this basis, Hudson and several other researchers have optimized the chemical decellularization method (Hudson et al., 2004; Yu et al., 2022; Topuz et al., 2022). Their improvements enable the successful removal of cellular components while minimizing damage to the ECM and preserving the structural integrity of the basement membrane. These advancements collectively provide a suitable environment for axonal regeneration.

In conclusion, when we screen decellularization methods, we should use milder treatments whenever possible to end up with neural scaffolds that do not disrupt the structure, composition, and function of the ECM. A representative improvement is to first treat with hypotonic or hypertonic solutions, followed by rinsing with mild nonionic or amphoteric detergents. If these methods still do not adequately remove cellular components, anionic decontaminants such as sodium deoxycholate and Triton X-100 should be used in the next step.

Despite years of research, the achievement of long-distance nerve regeneration using decellularized nerve grafts remains a formidable challenge. It is unclear which decellularization method strikes the optimal balance between removing cells, preserving natural neural architecture, and maintaining adequate biomechanical properties. Unfortunately, the lack of standardized evaluation methods makes it difficult to thoroughly compare different decellularization methods. Moreover, the pro-regeneration properties of decellularized grafts are also influenced by factors such as species, age, and gender. Therefore, future controlled studies incorporating well-designed experiments are necessary to elucidate the effects of different decellularization strategies on neural tissues.

#### Length of nerve defects

It is widely recognized that during repair of injury to peripheral nerves, the longer the nerve defect gap, the worse the prognosis for nerve regeneration (Peters et al., 2023). Even when nerve grafts of matched length are available for surgical repair, satisfactory functional recovery is usually difficult to achieve, and the phenomenon persists in clinical cases of nerve defect repair with decellularized grafts. Zhu et al. (2017) determined that the length of the nerve injury gap was an independent predictor of nerve repair using human decellularized allogeneic nerve grafts through a 3-year clinical follow-up study that analyzed 64 injured nerves. In patients treated with decellularized nerve grafts, functional recovery was more pronounced in the group with nerve gaps less than 30 mm than in the group with gaps greater than 50 mm. However, the exact reason for this phenomenon remains unclear. In this section, we provide a brief discussion of some of the possible mechanisms.

One possible explanation at the beginning of the study was that longer defects result in neurons that are more susceptible to senescence and death. Additionally, these neurons themselves possess limited intrinsic capacity for axonal regeneration, as difficulties in axons successfully spanning longer decellularized grafts are often observed in animal models and in the clinic (Whitlock et al., 2009; Vasudevan et al., 2014). However, several studies have suggested that the limited regeneration of long decellularized grafts compared to short decellularized grafts is due to the microenvironment that develops within the grafts rather than a defect in the neuron’s ability to regenerate axons over long distances (Pan et al., 2019; Lu et al., 2024).

*Vascularization*

It is widely accepted that vascularization during nerve regeneration is one of the necessary conditions for successful axonal regeneration. Newly created capillaries provide nutrients and oxygen for axonal growth and guide the direction of axonal growth. Previous studies have shown that cell-free scaffolds used for nerve repair reduce vascular regeneration compared to gold standard autografts, suggesting that the endothelial cell regeneration process of the scaffolds is inherently slow (Zucal et al., 2022; Smith et al., 2024). Thus, longer decellularized grafts may take longer to complete angiogenesis, predisposing to delayed or interrupted regeneration of blood vessels and thus impeding axonal regeneration. Interestingly, Acevedo et al. (2024) found that the density of regenerated blood vessels did not show significant differences in decellularized grafts of different lengths. However, for longer decellularized grafts, the morphology of the vessels within the mid-distal region of the grafts exhibited inflammatory pathology, including reduced vessel lumen and increased endothelial cell size, characteristic of the capillary thinning process.

*Schwann cells*

Axonal regeneration in decellularized grafts depends on the proliferation and support of the recipient’s own Schwann cells. Normally, SCs acquire a “repair” phenotype after nerve injury, downregulating myelin-forming genes and upregulating repair-related genes such as growth factors (Arthur-Farraj et al., 2012). However, in decellularized nerve grafts of different lengths, Schwann cells also appear to have their own specific phenotype. In short decellularized grafts, Schwann cells are more proliferative and express higher levels of myelin formation-related genes. In longer decellularized grafts, however, the Schwann cells express lower levels of myelin-forming genes but not high levels of repair-related genes. One possible mechanism contributing to the altered phenotype of Schwann cells is that delayed angiogenesis in long decellularized grafts may lead to a reduction in the number of entering Schwann cells and an altered phenotype.

In addition, more senescent Schwann cells were seen in long decellularized grafts. Saheb-Al-Zamani et al. (2013) first linked senescent cells in grafts to axon regeneration and found that restricted axon regeneration with increasing graft length was associated with an increase in senescent Schwann cells in the grafts. They found increased levels of senescence-associated markers (senescence associated β-galactosidase, p16INK4A, and IL6) in areas of axon growth arrest. Poppler et al. (2016) systematically investigated the growth and stagnation of axons within decellularized nerve grafts of varying lengths. They found that at 2 weeks post-transplantation, both short and long decellularized grafts were repopulated with large numbers of Schwann cells and stromal cells. Compared with short acellular grafts, long grafts accumulated higher levels of senescence markers and a higher percentage of Schwann cells expressing the senescence marker P16, significantly affecting axonal regeneration. In addition, when axon growth was arrested within a long decellularized graft, researchers excised a small segment from the distal end of this long graft that had not yet been invaded by axons. They then utilized this short segment of decellularized graft for repairing short neural defects. The results demonstrated restricted axon regeneration, thereby confirming that the newly formed microenvironment within the grafts plays a critical role in influencing the successful regeneration of axons. It is shown that there are differences in the microenvironment formed in long decellularized grafts versus short decellularized grafts.

*Immune cells*

Immune cells, such as macrophages, are the first cells recruited to the site of injury, and they repopulate the scaffolds of decellularized grafts, which play a key role in promoting nerve regeneration, especially in promoting angiogenesis (Takeuchi et al., 2022). Thus, disruption of immune cell proliferation or function can affect tissue regeneration. In one study, Pan et al. (2019) explored the differences in redistributed leukocytes in acellular grafts of different lengths. The results showed that the number of T cells within long grafts was significantly reduced compared to short grafts. These T cells secrete and regulate the levels of inflammatory cytokines within the decellularized grafts, and it is possible that through this regulatory mechanism, they mediate nerve regeneration across acellular grafts. During nerve regeneration, T-cell deficiency does not significantly impede axonal regeneration. However, in the absence of T cells during nerve repair using acellular nerve grafts, axonal regeneration and functional recovery were significantly compromised. This was manifested by a reduction in the number of myelinated axons, diminished axonal myelin formation, and attenuated functional recovery, all of which were associated with the absence of T cells.

To summarize, the length of decellularized grafts is negatively correlated with the ability to regenerate axons. Although the specific molecular mechanisms underlying this phenomenon remain to be fully elucidated, it is relatively clear that the localized microenvironment that re-forms within the graft plays a pivotal role in regulating and determining axonal growth in decellularized grafts. This microenvironment is related to the composition and phenotype of the cells that repopulate the grafts and involves vascular endothelial cells, Schwann cells, immune cells, and others. These cells have different fates in decellularized grafts of different lengths, resulting in different regenerative outcomes. In fact, other cells in the peripheral nerve are also involved in the regenerative repair of axons, including adipocytes, pericytes, and fibroblasts, and we hypothesize that these cells may also play some important roles in decellularized grafts of different lengths.

## Adverse Events Affecting Neurological Recovery

Blood vessels are hollow tubular structures whose primary function is to transport blood and surgical suturing or grafting can often directly restore their function as conduits for blood flow (Moritz et al., 2022). Unlike the cavernous structure of blood vessels, the nerves we see with the naked eyes are composed of many axons clustered together. These axons are protrusions and extensions of the neuronal cytoplasm, within which normal cellular physiological activities are carried out, including the transportation of substances and the transmission of signals. At the same time, a variety of cellular components are present in peripheral nerves, including Schwann cells, fibroblasts, endothelial cells, adipocytes, macrophages, and so on. These functionally distinct cells play a crucial role in the activities of peripheral nerves. They provide structural support and nourishment to the nerves, participate in the transmission of information between different tissues or organs, and regulate activities extending from the central to the peripheral nervous system. When a peripheral nerve is damaged, all the above-mentioned structures and components change and become involved in regeneration. As a result, the process of peripheral nerve regeneration is frequently quite complex, and surgical repair of peripheral nerves takes far more than simply reestablishing neural continuity. In fact, microsurgical repair of peripheral nerves has hit a plateau (Avache et al., 2024). Optimization of surgical techniques alone cannot significantly improve the recovery outcomes for patients with nerve injury.

Restoration of anatomical structural continuity is a prerequisite for nerve regeneration, while successful reinnervation of target organs by axons forms the basis for functional recovery. Numerous reported studies have demonstrated that damaged nerves undergoing the regeneration process can exhibit favorable parameters, such as the number and density of newly formed axons, remyelination, and myelin thickness (Gordon et al., 2024b; Xu et al., 2024a). However, these nerves often fail to achieve satisfactory outcomes in terms of final motor function recovery. These realities necessitate that researchers not only be satisfied with the successful reconnecting of transected nerves, but also focus on the process of reinnervation and repair after nerve grafting. By delving deeper into the regenerative mechanisms, researchers can potentially improve patient prognosis and alleviate the burden and suffering associated with nerve injury.

**Figure 9:**
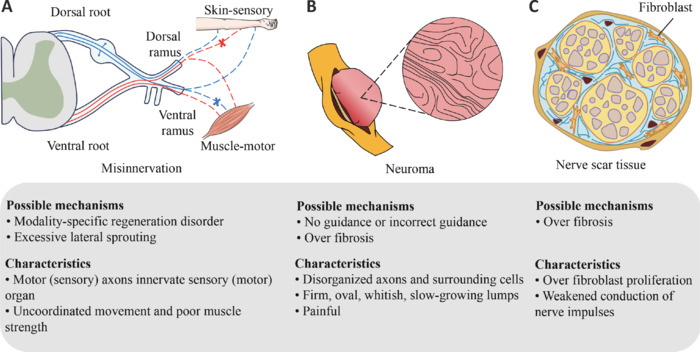
Adverse events associated with neurological recovery. (A) Misinnervation of new grown axons. (B) Neuroma. (C) Nerve scar tissue.

In this section, we briefly discuss several common phenomena that impede the prognosis of nerve grafts, including misinnervation of the target organ, neuromas, nerve scar formation, and immune response (**Figure 9**).

### Misinnervation

Neural regeneration is accompanied by the sprouting of newly grown axons, which will innervate target organs successfully only when the conditions are suitable (Lemaitre et al., 2021). Although many methods are currently being used to promote axonal regeneration, unfortunately, none of them can completely guarantee the success of the reinnervation process. Some of the most common types of mismatches include: (a) the inability of newly regenerated axons to accurately locate and connect with the corresponding target organs, and (b) the misalignment between sensory and motor fibers (**Figure 9A**). Any of these issues can result in reinnervation failure, which is a common cause for poor functional recovery.

One of the most common target organs innervated by peripheral nerves is muscle tissue (Adidharma et al., 2022). During nerve regeneration, muscle fibers that previously belonged to a particular motor unit are likely to be innervated by more than one motor axon. Also, a newly grown axon may connect with numerous muscle fibers at the same time (de Ruiter et al., 2014). Obviously, the remodeling of the structure of motor units may significantly alter normal movements or behaviors (de Carvalho et al., 2023). When the central nervous system gives commands to motor neurons, correct muscle regulation may not be achieved due to the mismatch between these neurons and the muscle fibers they actually innervate. For example, when the number of motor neurons innervating crucial large muscle groups is reduced due to mismatch or neuronal death, movement becomes significantly impaired (de Ruiter et al., 2008). Furthermore, when neurons that originally regulate the same muscles are each innervating to muscles with antagonistic functions, co-contraction of antagonistic muscle groups is likely to occur (Sumner et al., 1990). Another frequently cited type of abnormal innervation occurs when the axon of a sensory neuron innervates a motor unit, or the axon of a motor neuron innervates a sensory unit. In a study of nerve grafting in rodents, researchers have observed that when mixed nerves are transected and sutured above the bifurcation point of the sensory and motor nerves, following a period of regenerative repair, both motor and sensory neurons exhibit partly wrong projection (Brushart et al., 1993). This implies that motor nerve fibers may occupy the pathways that would normally be utilized by sensory nerve fibers to regulate cutaneous sensation, while sensory neurons may instead modulate the movement of muscles. Although these mis-projections can be self-recognized and pruned by themselves after a few weeks, a small fraction of this wrong innervation is still retained. A prolonged regeneration cycle will increase the risk of neuronal death and denervation atrophy of the target organ, leaving irreversible damage to the patient. A study has demonstrated that the transfer of a motor nerve with a higher axonal count into a target muscle, which was originally innervated by a motor nerve with fewer axons, can significantly alter the type and distribution of muscle fibers to the pattern observed in muscles innervated by the donor nerve. Concurrently, the strength of the target muscle is nearly fully restored to a level of motor function comparable to that of an uninjured state, a degree of recovery that is rarely achieved in cases of nerve transection injuries (Bergmeister et al., 2019). It is evident that successful reinnervation of the target muscle by corresponding and sufficient numbers of motor neurons is important for the recovery of motor function. However, several critical questions remain unresolved. It is still unclear whether the pruned axons will undergo regeneration again or simply die. Additionally, the extent of benefit that this pruning mechanism can provide in human patients remains to be determined (Allgood et al., 2023).

### Neuroma

Traumatic neuromas are not true malignant neoplasms but rather proliferative, reparative responses of nerve tissue to injury, often manifesting as nodular masses. These neuromas are typically characterized as firm, oval, whitish, slow-growing lumps composed of nerve fiber tissue and connective tissue. They are usually no larger than 2 cm in size and may be associated with altered sensation in the affected area (Yang et al., 2023; **Figure 9B**). They predominantly arise when normal nerve continuity is disrupted by injury, inadequate surgical repair, or chronic fibrous inflammatory stimulation (Oliveira et al., 2018). Any condition that may cause nerve damage may lead to the formation of a neuroma, and traumatic neuromas have been reported over a 10-year period following the use of different surgical methods such as radical cervical lymph node dissection, amputation, parotidectomy, abdominal surgery, orthognathic surgery and dental extractions (Foltán et al., 2008). Patients with nerve transection or amputations frequently develop painful neuromas at the site of nerve severance. Also, traumatic neuroma formation among patients undertaking nerve grafting is one of the most frequently reported disadvantages of peripheral nerve grafts. Although the incidence of postoperative symptomatic neuromas ranges from 1% to 30%, the exact incidence of post-injury neuroma formation remains unknown for there is no diagnostic or therapy for clinically asymptomatic neuromas (Huang et al., 2023; Zhou et al., 2023). The diagnosis of a neuroma is typically established based on a history of prior nerve injury or surgical intervention, coupled with clinical symptoms. These symptoms often include heightened pain sensitivity and the presence of trigger points that elicit nerve pain. Ultrasound and magnetic resonance imaging can be used to examine the anatomy and continuity of peripheral nerves to determine the location, extent, and type of injury, as well as for postoperative follow-up and complication detection.

Traumatic neuromas may form from abnormal axonal regeneration and organization following peripheral nerve injury or severance. Histologically, neuromas exhibit a disorganized, tangled structure comprising regenerating axons, connective tissues, and Schwann cells. This morphology suggests that the process of axon regeneration and their entry into the Bungner’s Band has been disrupted by certain factors. Factors such as slow regeneration speed, long nerve defects, and scar tissue can significantly impact the number of Schwann cells supporting axonal regeneration, the energy supply and activity of growth cones, and ultimately, the growth of axons. Additionally, inhibition of nerve growth factor or brain-derived neurotrophic factor has been shown to attenuate neuroma growth and alleviate neuropathic pain. Following injury, axons are capable of sprouting, and a single axon can concurrently maintain three to four lateral branches. When everything is correct, new grown axons can follow the guidance of Bungner’s Band toward the target organ, and this is regarded as ordered regeneration. However, some axons may lose control and grow in all directions without restraint, a condition known as disorganized regeneration, in which these scattered axons fail to develop distally and coil into neuromas (Fried et al., 1991). Though many researches have been reported, the exact molecular mechanisms and regulatory pathways underlying the formation of symptomatic neuromas remain unclear.

Currently, the most of research has focused on the surgical treatment of neuromas after their formation, as well as on surgical methods for the prevention of neuroma development (Langeveld et al., 2022). Neuromas that present with severe pain and symptoms typically necessitate surgical removal. Conservative treatment has been found to have a significantly lower success rate in such cases (Chou et al., 2023). However, not every type of neuroma is associated with severe symptoms, suggesting that the mere physical presence of a neuroma may not be problematic itself (Raasveld et al., 2024). For example, the proximal stump of a transected and non-immediately unrepairable nerve can be implanted into a muscle or vein, and the defect is subsequently repaired using decellularized nerve grafts or tissue engineering methods (Ederer et al., 2022). However, there is currently no consensus on the best technical approach to provide lasting benefits. The histopathology and electron microstructure of symptomatic neuromas possess several features, in which disorganized tangles of nerve fibers are the most prominent (Hwang et al., 2024). Changing disorder into order and directing targeted reinnervation of axons may be the key to avoiding some of the adverse prognostic events and promoting functional recovery after transplantation.

### Nerve scarring

Following nerve injury, the activation of regenerative pathways is accompanied by the initiation of healing and repair processes that aim to stabilize the anatomical integrity of the tissue. These processes involve a complex cascade of reactions that stimulate angiogenesis and fibroblast proliferation (Wang et al., 2019). While scar formation is a normal component of the repair process following tissue injury, excessive fibrosis that develops in response to nerve injury can impede normal axonal regeneration (**Figure 9C**). Extraneural scarring may compress the epineurium and whole nerve trunk at the injury site and limit its mobility, causing traction neuropathic pain. The pressure affects the tensile strength and elasticity of the nerve tissue which can lead to an 80% reduction in nerve diameter within three months post-injury in a case (Yannas et al., 2017). This alteration in biomechanical properties disrupts the normal biomechanical signaling within the nerve (Liu et al., 2021). Additionally, intraneural scarring occurs within the parenchymal structure of the nerve and may cover anything inside the epineurium, including the vessels and axons. It weakens the microcirculation of the nerve, leading to further ischemia and secondary axonal degeneration (Servet et al., 2016). These scars tend to extend within the transected nerve and the extent of scarring is correlated with the severity of the nerve injury (Crosio et al., 2021). The transforming growth factor-β family (TGF-β) plays a prominent role in wound healing and fibrosis and its isoforms have different biological potencies and actions respectively (Chen et al., 2024; Deng et al., 2024). TGF-β1 exerts chemotactic effects on macrophages and fibroblasts, stimulates angiogenesis and granulation tissue formation, and modulates extracellular matrix dynamics by promoting its synthesis and inhibiting degradation. In contrast, TGF-β2 is less potent but may synergistically interact with TGF-β1. Anyway, scarring at the site of nerve injury severely restricts the optimal recovery of nerve function and continues to pose a significant and unresolved clinical challenge.

Current strategies to prevent and reduce the scar tissue in nerves are diverse. First, restoration of continuity in a transected nerve serves as the fundamental approach to minimize scar formation. Tight and precise suturing of the nerve ends and the utilization of artificial adhesives have been demonstrated to significantly reduce the incidence of scarring (Suri et al., 2002). Second, nerve conduits and other natural or artificial membrane grafts can cover and create an isolated environment at the injury site that minimizes infiltration by fibroblasts and inflammatory cells, thus reducing fibrosis around the nerve (Li et al., 2018). Third, a number of drugs and chemical compounds have been developed to prevent fibrosis and promote axonal growth, including corticosteroids, glycosaminoglycans, calcium antagonists, hyaluronic acid, melatonin, and others (Occleston et al., 2008; Zujidendorp et al., 2008; Atik et al., 2011; Li et al., 2016a; Xue et al., 2016; Mekaj et al., 2017). Furthermore, it has been reported that low-dose local radiation and early rehabilitation exercises may also be effective (Gocmen et al., 2012).

### Immune response

One of the most common and serious adverse effects following tissue transplantation is immune rejection, which often leads to the failure of transplantation therapy. Among the various nerve transplantation methods, autologous nerve transplants are typically well-adapted immunologically and are currently regarded as the gold standard for achieving optimal therapeutic outcomes. In contrast, allogeneic nerve transplants require long-term administration of immunosuppressive drugs. These drugs can be toxic and are associated with side effects such as opportunistic infections, an increased risk of diabetes, malignancy, and renal failure, making the use of allogeneic nerve grafts less common in clinical practice (Parlakpinar et al., 2021; Wojciechowski et al., 2021; Russo et al., 2024).

Homologous nerves are highly immunogenic. However, studies have shown that the immunogenicity of nerve extracellular matrix components, such as laminin and fibronectin, is minimal to negligible (Gao et al., 2013; Xu et al., 2024b). Consequently, the immunogenicity of homologous nerves is primarily attributed to their cellular constituents (Li et al., 1998; Berner et al., 2022). Notably, Schwann cells and myelin sheaths are recognized for their pronounced immunogenicity (Evans et al., 1997). Schwann cells, a major cellular component of peripheral nerves, have an antigen-presenting function and produce cytokines such as interleukin-1 and interferon-γ, which can induce immune rejection (Lilje et al., 1997; Brenner et al., 2005). Additionally, the MHC II molecules on the surface of Schwann cells are the primary antigenic substances responsible for the immune rejection of allogeneic transplants (Boucher et al., 2007). Rejection of transplanted tissue is typically triggered by a localized innate inflammatory response, which enhances the subsequent adaptive response. Macrophages and dendritic cells migrate into the grafts, initiating an inflammatory response and recruiting adaptive immune cells, such as T cells. These T cells release inflammatory factors, chemokines, and reactive oxidants that exacerbate tissue damage. In some cases, immediate congenital rejection of allogeneic grafts can occur rapidly due to preexisting blood or polymorphic MHC antigen-specific alloantibodies, a phenomenon known as hyperacute graft rejection. Once this process begins, it is challenging to halt, leading to complete destruction of the allograft tissue. Interestingly, several studies have indicated that peripheral nerve allografts can promote nerve regeneration to some extent without the need for immunosuppressive therapy. This contrasts with the transplantation of other organs or tissues, which typically requires the administration of immunosuppressive agents to prevent rejection. Macrophages accumulate during the first 2–3 days post-injury, which may correlate with their important role in promoting regeneration (Mokarram et al., 2017). Although the initial immune response was more pronounced in autografts compared to allografts, this response diminished over time. Surprisingly, effector T cells mediating acute graft rejection did not peak in allogeneic nerve grafts as they do in skin and other tissues, indicating a degree of immune privilege for peripheral nerves. Even in the absence of immunosuppression, the extent of CMAP and muscle mass recovery were very similar between autografts and allografts, with the differences gradually decreasing over time. The study also demonstrated comparable abundance and localization of host immune cells in both autologous and allogeneic nerve grafts.

However, the mechanism by which allogeneic nerve grafts are superior to other tissue organ grafts for this immune privilege is unknown. One potential explanation is that the membrane structure of peripheral nerves slows the infiltration of peripheral immune cells (Klein et al., 2016). This is because the epineurium has not only a dense basement membrane composed of type I collagen fibers, but also a blood–nerve barrier composed of connective tissue and vascular system (Maiuolo et al., 2019). Surprisingly, it has also been observed that immune cells infiltrate into the graft mainly from the nerve suture site rather than along the outer surface of the nerve (Mokarram et al., 2017). Therefore, additional research is still necessary to elucidate the sources of immune infiltration in allogeneic nerve grafts and to quantify the extent of their contributions. Another potential explanation is that acute rejection of allogeneic tissues is primarily mediated by disparities in MHC and minor histocompatibility antigen expression (Nardo et al., 2016). In peripheral nerves, however, baseline MHC expression is lower compared to other tissues. Neurons are one of the few classes of cells that express little or no MHC I at steady state and may represent a relatively immune-privileged cell population. After allogeneic nerve transplantation, both MHC I and MHC II, although proportionally increased, were not as robust as those observed in skin grafts. In conclusion, allogeneic peripheral nerves are functionally and immunologically unique from many other transplanted allogeneic tissues.

In contrast to allogeneic nerve grafts, which may elicit immune rejection, the rapid advancement of decellularized grafts has provided novel avenues to address this challenge. One of the most prominent advantages of decellularized nerve grafts is the removal of tissue components that trigger a host response, thereby removing the immunogenicity of the graft. Consequently, immune rejection is generally not observed in decellularized grafts that have been successfully engineered. It is only when cellular debris or myelin remains in the decellularized extracellular matrix and is not completely removed that a certain inflammatory response is triggered, leading to destruction of the graft or formation of scar tissue. Additionally, the damage-associated molecular pattern (DAMP) proteins are generated as a result of host cell death and cell membrane disruption caused by the detergents utilized in the decellularization process (Badylak et al., 2014). DAMPs molecules possess proinflammatory, chemotactic, proliferative, and tissue regenerative properties. Consequently, residual DAMPs, like other cellular residues, are highly likely to influence the host’s immune response to the extracellular matrix.

## Safety and Biocompatibility

The safety of autologous or allogeneic nerve grafts is primarily associated with immune rejection, as discussed in previous sections. In this section, we will focus on the safety and biocompatibility of decellularized grafts. Generally, decellularized tissues or organs are widely regarded as some of the most biocompatible and non-cytotoxic scaffolds for various tissue engineering applications. These decellularized scaffolds can be directly utilized in clinical settings as medical devices, such as porcine or sheep heart valves (Inal et al., 2024; Solecki et al., 2024; Martínez-López et al., 2025).

### Safety

Decellularized nerve grafts, as one of the promising clinical methods for repairing nerve defects, need to be clarified in terms of their safety and efficacy before they can be translated into the clinical setting. Unlike artificial nerve conduits that are manufactured using a variety of synthetic materials, both natural nerve grafts and decellularized nerve grafts are fundamentally derived from the natural tissue structures of living organisms. As such, they do not typically induce significant biological or cytotoxic effects themselves. However, tissues and organs undergoing decellularization are exposed to different kinds of chemical, biological or physical reagents that may have different effects on the biocompatibility of these scaffolds. In this case, the main risk may be related to inadequate cleanup of chemical residues from the decellularized scaffold (Kraft et al., 2020). These residual detergents may lead to cytotoxicity and trigger inflammatory responses, thereby inhibiting cell adhesion and proliferation (Klak et al., 2021; Guo et al., 2024a, b). Therefore, extensive washing steps are necessary to thoroughly remove decontaminants and residual chemicals, thereby minimizing their levels and preventing cytotoxicity and degradation of the ECM (Naso et al., 2022; Hussein et al., 2024).

Inadequate decellularization can lead to safety issues, such as inflammatory reactions. To date, there are no comprehensive and universally accepted criteria or procedures for evaluating the degree of decellularization of tissues. Crapo et al. (2011) proposed objective criteria for assessing the effectiveness of decellularization based on years of experience. These criteria include: (1) the absence of nuclei as determined by histologic staining with hematoxylin and eosin and 4′,6-diamidino-2-phenylindole; (2) quantitative measurements of DNA content below 50 ng/mg; and (3) DNA fragment sizes less than 200 bp. Grafts that meet these criteria are often considered safe and effective because residual DNA can trigger an inflammatory response in the recipient, compromising the integrity of the scaffold (Emami et al., 2021). However, it is important to note that these criteria primarily reflect the evaluation of denuclearization rather than complete decellularization. Some studies assessing decellularized grafts using these criteria have found that effectively decellularized and ineffectively decellularized tissues exhibit similar host remodeling responses (Keane et al., 2012; Manon et al., 2025). This finding suggests a potential limitation in accurately assessing the efficacy of decellularization using the proposed criteria. Additionally, van Hengel et al. (2025) provide reference criteria for evaluating the safety of decellularized tissues from multiple perspectives in detail. Overall, the reliability of existing evaluation criteria needs to be confirmed through further experimental studies.

### Biocompatibility

Biocompatibility is one of the most important considerations before decellularized grafts are put into clinical use. Good biocompatibility facilitates the integration of the graft with the surrounding tissue and promotes cell migration and proliferation, thereby enabling successful axonal regeneration. Poor biocompatibility may trigger a variety of adverse reactions in the body, impede successful nerve regeneration, and cause injury to the recipient. The biocompatibility of decellularized grafts involves two main issues, whether the graft will produce a severe inflammatory response after transplantation and whether the scaffolding structure of the graft can effectively support axonal regeneration and repair.

The inflammatory response is primarily related to residual cells, myelin debris, and biochemical reagents in decellularized grafts. Residual chemical and biological reagents enter the host with the graft and amplify their reagent toxicity by osmosis or blood transport. Physical decellularization only destroys cells to reduce their immunogenicity, but does not remove these fragments from the extracellular matrix. After transplantation, Schwann cells, macrophages, and other inflammatory cells rapidly migrate into the graft, engulfing the debris and initiating an inflammatory response that causes degeneration and degradation of the graft. One study clearly demonstrated that greater retention of double-stranded DNA, as an indicator of “cellular debris,” was associated with a significantly enhanced pro-inflammatory response and adverse downstream remodeling responses (Keane et al., 2012). Different degrees of inflammatory responses may have different effects on axonal regeneration. Appropriate inflammation accelerates vascular regeneration and facilitates axonal growth, whereas excessive inflammatory responses cause damage to scaffold structures and even trigger scar formation, which hinders axonal growth. Therefore, the removal of residual cellular components from the ECM helps to mitigate the inflammatory response, thereby enhancing biocompatibility.

Decellularized grafts preserve the natural ECM and laminin components of the nerve, providing a suitable regenerative 3D biological scaffold, which is not possible with any reported artificial nerve conduits. When the graft scaffold is destroyed, its structural superiority cannot be fully realized. Therefore, preserving the natural structure to the greatest extent possible is essential for achieving good biocompatibility. Nevertheless, no existing decellularization method can ensure the complete removal of all cellular components without disrupting the ultrastructure and ECM composition of the tissue. For example, sodium dodecyl sulfate and other ionic detergents are excellent at removing cytoplasmic and nuclear material, but have a tendency to disturb the natural structure of the tissue by disrupting protein-protein interactions (Seddon et al., 2004). Therefore, when refining a decellularization strategy or obtaining a new decellularized tissue, a thorough and rational assessment of the integrity of its scaffold should be conducted. Qualitative and quantitative evaluation methods encompass histology, ultrastructural analysis, biochemical assays, and biomechanical testing. It is essential to confirm the cell-free scaffolds retain the natural three-dimensional structure without significantly altering the composition of the ECM. Additionally, these scaffolds should possess biomechanical properties comparable to those of native tissues in order to support cell proliferation and axonal regeneration.

Therefore, qualitative and quantitative evaluation methods, including histologic, ultrastructural, biochemical, and biomechanical analyses, should be performed for successfully decellularized grafts. It is necessary to confirm that the obtained cell-free scaffolds maintain the natural three-dimensional tissue structure without severely affecting the composition of the ECM and have biomechanical properties comparable to those of natural tissues to support cell proliferation and axonal regeneration. The specific methods and strategies for assessing the efficiency of decellularization and the structural integrity of nerve grafts following decellularization are beyond the scope of this article. These topics are comprehensively reviewed in the work described by Philips (Philips et al., 2018).

Currently, one of the simplest methods to assess safety and biocompatibility is to culture cells on the surface of the decellularized neural grafts, and then record and analyze parameters such as cell activity, morphology, and proliferation, thereby clarify whether the decellularized grafts are safe and effective. In addition, acellular nerve grafts inoculated with cells can also be subjected to histochemical and immunohistochemical analyses. These analyses can provide information on the distribution of cells after inoculation and can be used to confirm whether the cells can migrate through the decellularized scaffold (Roosens et al., 2017). It is important to note that the number of cells may decrease again after a period of incubation due to contact inhibition or reduced viability and functionality in the culture (García-Martínez et al., 2017).

## Clinical Advances and Translation

It has been proposed that, in the future, autologous nerve grafts may no longer serve as the gold standard for the clinical treatment of peripheral nerve deficits, and instead, decellularized grafts and their derivatives may emerge as viable alternatives. Upon reviewing the clinical development of decellularized grafts, we are convinced that their potential applications and contributions in the future appear to be boundless.

We focus our discussion on registered clinical studies and their related reports. We searched the International Clinical Trials Registry Platform (ICTRP) and ClinicalTrials.gov (CTG) for relevant registered clinical trials, and included clinical trials evaluating the effects of decellularized nerve grafts on nerve repair in this study. Studies with the status of “not yet recruited” and studies not related to regenerative repair after nerve injury were excluded. Relevant clinical studies are shown in **Additional Table 3**.

**Additional Table 3:** Overview of clinical trials registered in ICTRP or CTG for the treatment of peripheral nerve injury using decellularized tissue

Registration number, Status (study duration)	Condition	Study title	Study overview	Product name	Source	Region	Decellularization method	Reference
NCT00948025, Terminated (2009-2020)	Digital nerve injury, laceration/sharp transection	A comparative post marketing study of commercially available peripheral nerve gap repair options	Sensory recovery outcomes: AVANCE graft *vs*. hollow tube conduit for hand nerve gap repairs	Avance nerve graft	Human peripheral nerve	USA	50 mM phosphate + 100 nM Na, 0.14% Triton X-200, 0.6 mM sulfobetaine-16	Means et al., 2016; Safa et al., 2020
ChiCTR-TNRC-11001665, Completed (2009-2017)	Digital nerve injury	Human acellular nerve graft for repair of peripheral nerve defects: a prospective, multicentre clinical study	To investigate the safety and efficacy of human acellular nerve graft for the repair of peripheral nerve defects.	Human acellular nerve graft	Human peripheral nerve	China	46 mM Triton X-100, 96 mM sodium deoxycholate	He et al., 2015; Kasper et al., 2020
ChiCTR-TNRC-11001443, Phase 3, Completed (2009-2017)	Digital nerve injury	Human acellular nerve graft for repair of pure sensory nerve defects: a prospective, multicentre clinical study	To investigate the safety and efficacy of human acellular nerve graft for the repair of peripheral nerve defects.	Human acellular nerve graft	Human peripheral nerve	China	46 mM Triton X-100, 96 mM sodium deoxycholate	He et al., 2015; Kasper et al., 2020
NCT01809002, Phase 3, Completed (2015-2021)	Peripheral nerve injury of upper limb	Comparison of processed nerve allograft and collagen nerve cuffs for peripheral nerve repair (RECON)	Processed nerve allograft and collagen nerve cuffs will be compared to assess safety and functional outcomes for the repair of nerve injuries in the hand	Avance nerve graft	Human peripheral nerve	USA	50 mM phosphate + 100 nM Na, 0.14% Triton X-200, 0.6 mM sulfobetaine-16	Lacs et al., 2023
NCT03964129, Phase 1, Unknown status (2017-2021)	Peripheral nerve injury of upper limb	BMAC nerve allograft study	Phase I study evaluates safety of Avance nerve graft + BMAC for repairing nerve injuries up to 7 cm. Aims to support combining stem cells with scaffolds.	Avance nerve graft	Human peripheral nerve	USA	50 mM phosphate + 100 nM Na, 0.14% Triton X-200, 0.6 mM sulfobetaine-16	No related report
NCT01526681, Recruiting (2008-)	Peripheral nerve injury	Registry of Avance® Nerve Graft's utilization and recovery outcomes post peripheral nerve reconstruction (RANGER®)	This study is a registry of Avance nerve graft's general use, evaluating its real-life clinical applications, response rates, and safety.	Avance nerve graft	Human peripheral nerve	USA	50 mM phosphate + 100 nM Na, 0.14% Triton X-200, 0.6 mM sulfobetaine-16	Rinker et al., 2017; Safa et al., 2019; Safa et al., 2020; Van et al., 2024

ICTRP: International Clinical Trials Registry Platform; CTG: ClinicalTrials.gov; BMAC: bone marrow aspirate concentrate.

Among the registered clinical studies that met the requirements, we found that there are two main types of decellularized nerve grafts used, one is the only FDA-approved commercially available Avance ® Nerve Graft (AxoGen, Inc), which is mainly marketed in the United States and used for clinical treatment. The other is human acellular nerve grafts (hANGs) (Guangzhou Zhongda Medical Devices Company, Guangzhou, Guangdong Province, China), with the Chinese name of “Shen Qiao”, which is mainly marketed and used for clinical treatment in China. We will discuss the use of these two commercially available decellularized nerve grafts separately (**Additional Table 4**).

**Additional Table 4:** Comparison of selected hANGs and AVANCE clinical study reports

	hANGs		Avance nerve graft			
	
	He et al., 2015	Zhu et al., 2017	Means et al., 2016	Karabekmez et al., 2009	RECON (Isaacs et al., 2023)	RANGER (Safa et al., 2020)
Condition	Digital nerve injury	Peripheral nerve injury of upper limb	Digital nerve injury	Peripheral nerve injury of upper limb	Digital nerve injury	Peripheral nerve injury
Nerves	72	64	18	10	112	624
Follow-up	Data not available	355 ± 158 (35-819) d	6-12 mon	9 (5-12) mon	12 mon	417 (120-3286) d
Age (yr)	33.0±11.1 (18-61)	35±11 (14-68)	42±13 (21-63)	44 (23-65)	36±13.6 (18-68)	41.59±16.73 (6-83)
Time from injury to repair	23.7±52 (0-200) d	Data not available	Data not available	Data not available	< 24 wk	Median: 2 (0-4452) d
Length (mm)	18.0±8.2 (10-50)	27±13 (10-60)	12.8±4.6 (5-23)	22.3 (5-30)	5-25	24±15 (3-70)
Outcome	s2PD: 65.28% (excellent and good) (95% CI was 51.98%- 78.93%)	75% experienced a meaningful sensory or motor recovery	Average s2PD: 5±1 mm Average moving 2PD: 5±1 mm	Average s2PD: 5.5 mm Average moving 2PD: 4.4 mm	Average s2PD: 7.3±2.8 mm (GAP 5-14 mm), 6.1±3.3 mm (Gap 15-25 mm)	Cumulative meaningful recovery was reported in 82% of these repairs.
Adverse events	No	No	Infection: 1 case	No	2 cases	No

hANGs: Human acellular nerve grafts; PNGs: processed nerve grafts; s2PD: mackinnon-dellon static two-point discrimination. RECON: The first registered clinical trial comparing Avance nerve graft and nerve conduits. RANGER: The first and largest registered clinical trial using Avance nerve graft (began in 2008 and is still ongoing).

### Human acellular nerve grafts

We previously assumed that all clinical studies on nerve repair using decellularized nerve grafts would necessarily involve the Avance Nerve Graft, as it is the only one approved by the FDA for marketing. Therefore, it was intriguing to discover the registered clinical studies related to hANGs (Yang et al., 2011; He et al., 2015). The existence of hANGs indicates that researchers worldwide are actively engaged in efforts to identify suitable nerve replacements. These grafts offer an alternative strategy for preparing decellularized grafts for clinical use. The clinical application of hANGs has been thoroughly investigated over an extended period.

The preparation, histological characterization, and biocompatibility of hANG were described in detail by Yang et al. (2011). This approach is a modified version of the Sondell method. In brief, the nerves were stirred in deionized distilled water for 6 hours, after which the water was replaced with a distilled water solution containing 46 mM Triton X-100. Following 24 hours of agitation, the scaffolds were rinsed three times with a 10 mM phosphate-buffered saline solution. The nerves were then agitated in a 96 mM sodium deoxycholate solution dissolved in deionized distilled water for an additional 24 hours. All processing steps were conducted at room temperature, and the scaffolds were subsequently stored in 10 mM phosphate-buffered saline at 4°C. The researchers designed and conducted tests to evaluate the *in vitro* cytotoxicity, hemolytic potential, and skin sensitization of hANG. Their results confirmed that the chemically treated human nerves do not induce cytotoxicity, hemolysis, or skin sensitization, demonstrating their safety for *in vivo* implantation.

He et al. (2015) were the first to explore the safety and efficacy of hANGs in a study involving 72 human patients. They assessed safety through local wound responses and laboratory tests. At the one-month postoperative follow-up, all patients exhibited grade II/A healing for contaminated wounds and grade I/A healing for clean wounds. Laboratory findings were normal in all patients, except for two cases with slight elevations in liver enzymes. At one month postoperatively, no patients reported pain, itching, localized erythema, urticaria, rash, or other allergic symptoms, and no currently known infectious agents were detected. The hANGs demonstrated an excellent rate of repairing nerve injuries, ranging from 51.98% to 78.93%. This clinical trial confirms the safety and efficacy of hANGs in humans and offers valuable insights for subsequent research endeavors. Zhu et al. (2017) further investigated the use of hANGs for repairing nerve injuries in the hand and upper extremity, examining 64 cases over a 3-year follow-up period. In this study, they expanded the scope to include peripheral nerves of the hand and upper extremity, suggesting that hANGs could safely and effectively reconstruct nerve defects ranging from 10 to 60 mm in length, achieving a meaningful recovery rate of 75%. Factors such as the site of the injury and defect length significantly affected the outcomes of nerve reconstruction, with superior results observed in the group with a gap length of 30 mm compared with the group with a gap length of 50 mm or more (87.8% *vs.* 33.3%).

### Avance nerve graft

The Avance Nerve Graft is currently the only commercially available decellularized nerve graft approved by the FDA (Kasper et al., 2020). It uses a modified strategy based on the Hudson method and was commercialized in 2007. The Avance Nerve Graft addresses the issue of donor-site morbidity associated with autologous nerve grafts while providing a three-dimensional microstructural scaffold and a protein composition intrinsic to nerve tissue. These grafts effectively overcome the problem of immune rejection while preserving the integrity of the extracellular matrix (ECM). Additionally, the internal structures of the nerve epineurium and endoneurial canal are preserved.

The first clinical study utilizing the Avance Nerve Graft was reported by Karabekmez et al. (2009). They employed decellularized nerve grafts to treat sensory nerve defects and found that all 10 treated nerves successfully fused with the repair site, allowing for adequate sensation to be restored in nerve gaps ranging from 0.5 to 3 cm. No adverse symptoms, such as infection or rejection, were observed during the follow-up, demonstrating the practical clinical effectiveness of the Avance Nerve Graft and providing a novel option for the clinical repair of nerve injuries. Means et al. (2016) compared the outcomes of processed allogeneic nerve grafts with nerve conduits for digital nerve reconstruction in the hand. In this preliminary study, a total of 31 damaged nerves were repaired, all of which had gaps ranging from 5 to 20 mm, with no symptomatic neuromas reported. Patients who underwent digital nerve reconstruction using the Avance Nerve Graft experienced significantly improved sensory outcomes compared to those who received nerve conduits.

Additionally, two notable clinical trials of particular relevance to the Avance Nerve Graft are the RECON and RANGER trials (Rinker et al., 2017; Isaacs et al., 2023). The RECON study is a Phase III trial focused on a change in regulatory classification. It aims to compare the clinical application of the Avance Nerve Graft with that of the Collagen Nerve Cuff conduit for the repair of hand nerve defects ranging from 5 to 25 mm. The primary objectives of this study are to evaluate safety and patient functional recovery outcomes, using static two-point discrimination at 12 months postoperatively as the main evaluation criterion. In 2023, the RECON study team published their clinical report online (Isaacs et al., 2023). They found that digital nerves repaired with the Collagen Nerve Cuff showed similar levels of regeneration compared to those repaired with the Avance Nerve Graft in the short gap length group (5–14 mm). However, in the long gap length group (15–25 mm), the Avance Nerve Graft demonstrated clinical superiority over nerve conduits. This study has important implications, as both nerve conduits and decellularized nerve grafts exhibit significant clinical potential and serve as promising alternatives to autologous nerve grafts. The findings clearly elucidate the strengths and limitations of these two widely used materials, providing valuable guidance for clinical treatment options. Moreover, this direct comparison has heightened enthusiasm for further research into these two approaches. While the synthesis of bionic nerve conduits using various materials is currently a research hotspot, no conduit has yet truly matched the natural scaffold provided by decellularized grafts. The superior clinical performance of decellularized grafts is likely to drive further research in nerve conduit development.

The RANGER study is a multicenter observational registry designed to gather real-world experience with surgical nerve repair using the Avance Nerve Graft. It is the largest single clinical study of peripheral nerve repair to date. The study began in 2008 and is still ongoing, actively monitoring and collecting data on nerve injuries, repairs, safety, and outcomes across various injury modalities and body regions. To date, the RANGER study has enrolled approximately 5000 patients, and researchers have published multiple peer-reviewed clinical publications detailing their analyses of the database (Rinker et al., 2017; Safa et al., 2019; Van et al., 2024). The latest interim report from 2020 includes data on 385 subjects with 624 nerve repairs (Safa et al., 2020). Digital nerve repairs using the Avance Nerve Graft demonstrated a meaningful recovery rate of 83%, compared to literature estimates of approximately 70% for autografts, highlighting its effectiveness in nerve repair. The effective repair rates for sensory, mixed, and motor nerves were 84%, 71%, and 83%, respectively. Notably, the percentage of meaningful recovery was significantly higher in upper extremity nerve repairs (83%) than in lower extremity repairs (53%, *P* < 0.05), which is consistent with findings from autografting in clinical settings. The most common adverse events observed were neuromas at the repair site, with an incidence of 1.2%, followed by infections at the repair site, with an incidence of 0.9%. None of the adverse events were attributed to the product itself; rather, they were related to the environment surrounding the original injury. There were no reports of infectious disease transmission. Overall, the adverse event rates observed are consistent with expected rates for surgical procedures in the United States. These findings demonstrate that the use of processed nerve grafts is safe and suggest that the Avance Nerve Graft poses no additional risk to patients.

## Limitations

This study has several limitations. First, the lack of standardized assessment methods presents significant challenges when attempting to make comprehensive comparisons among various decellularization techniques. Factors such as species, age, and gender can significantly influence the pro-regenerative potential of decellularized grafts. Therefore, future studies that incorporate well-designed experiments are essential to clarify the effects of different decellularization strategies on neural tissues. Second, due to uncertainties surrounding the factors and mechanisms by which grafts influence nerve regeneration, and considering the constraints on article length, we have chosen to emphasize key findings rather than provide exhaustive details on all potential factors.

## Conclusions and Perspectives for the Future

The field of nerve grafting and repair research has a long and storied history, marked by ongoing exploration of new methods to promote nerve regeneration. Unfortunately, the prognosis for patients undergoing grafting in clinical practice is often not significantly improved, with many still requiring a second surgery. These patients face risks of lifelong pain, varying degrees of paralysis, and even amputation. While the consequences of peripheral nerve failure may not be as fatal as those associated with central nervous system failure, they nonetheless impose a substantial burden on both patients and society. Peripheral nerves possess a regenerative capacity that surpasses that of the central nervous system, making it the responsibility of surgeons and researchers to identify rational approaches that maximize this repair ability for the benefit of patients. Drawing on our clinical experience and previous research, we would like to present some future perspectives on peripheral neurosurgical repair.

(1) The cellular components of peripheral nerves are crucial to nerve graft repair. It is important to note that the nerve fibers in peripheral nerves are not the neurons themselves, which are located in the dorsal and ventral roots; rather, they are extensions of the neuronal cytoplasm. The regeneration of damaged nerves involves axonal regrowth to reach and reinnervate the target organ, a process that is significantly influenced by the nerve grafts used to repair the severed ends of the nerves. The combination of decellularized grafts with individual cellular components has produced favorable repair outcomes, highlighting the importance of these cellular elements for axonal regeneration. To enhance the efficacy of grafts and fully harness their pro-regenerative effects on nerve repair, future studies should focus on the cellular components present in grafts or transection injury models. This includes investigating Schwann cells, endothelial cells, macrophages, and fibroblasts. Examining these cells at different stages and states, along with their functions through advanced technologies such as various omics approaches and lineage tracing, will facilitate the more efficient and personalized use of cellular components. This approach aims to promote the recovery of neurological function after transplantation in the future.

(2) Grafts of sensory and motor nerve origin contribute differently to nerve repair. Autologous nerve grafts, which are considered the gold standard in neurosurgical repair, often employ pure sensory nerves as graft materials to reestablish continuity in severed nerves, with the goal of regaining some of motor function. However, such therapies typically produce inferior clinical outcomes. In rodent models of nerve grafting, motor nerve grafts have been demonstrated to promote mixed nerve regeneration better than sensory nerve grafts. However, when repairing purely sensory or purely motor nerves, grafts derived from these two distinct nerve sources have been shown to exhibit no significant differences in terms of functional recovery or regenerative outcomes. These findings indicate that there are differences in the regenerative capacity of sensory, motor and mixed nerves, suggesting that we need to select appropriate grafts according to the type of injured nerve when performing nerve graft repair. Currently, there are few studies related to the differences between motor and sensory nerves in nerve regeneration and repair. We think clarifying the differences and mechanisms between these two types of nerves and their grafts in nerve repair will help to achieve specialized and specific restoration of the function of injured nerves, as well as provide a basis for decision-making on nerve grafting in clinical patients.

(3) Good histological results do not guarantee good functional outcomes. Histological data on nerve regeneration, including parameters such as axonal density, fiber count, and myelination, often do not correspond to the actual level of functional recovery. Moreover, as the duration of animal experiments increases, the final outcomes of functional recovery tend to converge. This intriguing phenomenon warrants further exploration. Theoretically, histologic regeneration forms the basis of functional regeneration, so clarifying the mechanisms that link histology and function may be key to improving clinical outcomes. Additionally, there is a need for a uniform and comprehensive standard for evaluating recovery after nerve injury. As mentioned previously, the uncertain relationship between histologic regeneration and functional recovery undermines the credibility of conclusions from some earlier studies that were limited by advanced motor or sensory function analysis techniques. Furthermore, because peripheral nerve tracing techniques can visually demonstrate the structural continuity of axons and the functional integrity of material transport within them, we suggest that these tracing techniques be used as one of the criteria for evaluating nerve regeneration, especially when there is a significant discrepancy between histological and functional outcomes. Ultimately, the final result of functional regeneration is the most critical factor in the recovery of injured nerves.

(4) Transplantation experiments should be conducted by selecting appropriate experimental animals and models. Currently, most transplantation experiments are performed in rodents or rabbits, and these animals have significantly contributed to the advancement of nerve transplantation research. However, small animals demonstrate superior nerve regeneration and repair capabilities compared to humans (Griffin et al., 2010). As a result, it is often easier to observe functional recovery in rodent nerve transplantation studies that resemble the outcomes achieved with autologous nerve grafts. This finding is clearly inconsistent with actual clinical practice, where such reliable and informative recovery results are rarely achieved. Therefore, we believe that, if possible, nerve grafting experiments should utilize larger animals, such as pigs, dogs, or even nonhuman primates, as study subjects.

(5) Gene editing or engineered modification of peripheral nerve grafts has emerged as a significant area of research. Xenotransplantation to humans using internal organs from gene-edited animals has gained attention over the last decade; however, this method requires gene editing of the entire animal and is complicated, expensive, and time-consuming. With continuous advancements in drug delivery, *in situ* gene editing of specific target organs in living animals using viruses, lipid nanoparticles, and other vectors has become feasible. In fact, tissue gene editing of peripheral nerve graft fragments is both highly feasible and significant.

First, peripheral nerve graft fragments differ from vital internal organs such as the heart, liver, and kidney. They are characterized by their relatively small size (typically a few centimeters), rapid vascular regeneration, well-defined cellular composition, high regenerative capacity, and relatively straightforward surgical transplantation procedures. Second, Schwann cells, which are essential for nerve growth, development, and repair, have the ability to spontaneously alter their phenotype to regulate and direct axonal regeneration. This phenotypic plasticity can be modulated by specific treatments, thereby influencing the outcomes of nerve repair and regeneration. Based on these characteristics, we propose that gene editing technology could be employed to enhance the activity of nerve graft fragments and create a more favorable environment for nerve regeneration. There are two primary approaches under consideration. The first involves direct *in situ* gene editing of nerve graft segments, where researchers can utilize in situ injection within the perineurial membrane to deliver the gene-editing agents. This method significantly simplifies the drug delivery process, enhances the localized gene editing effect, and minimizes the potential impact on other tissues and sites. The second approach involves combining cellular components that have undergone gene editing with decellularized grafts to construct novel nerve grafts. This method is not restricted by species origin and offers the potential for long-segment allogeneic nerve grafts, thereby expanding the scope of nerve repair strategies. Given the significant breakthroughs and advancements in gene editing technology within the field of organ transplantation, we posit that applying gene editing modifications to peripheral nerve grafts represents a personalized and highly promising therapeutic strategy for improving the final outcomes of neurological restoration.

(6) Decellularization methods need to be continuously updated. Despite the availability of commercial decellularized nerve grafts that have demonstrated promising outcomes in clinical settings, these grafts are predominantly derived from modifications of the Sondell & Hudson method, a technical approach that originated nearly two decades ago. Strong chemical reagents can effectively remove cellular components and myelin sheaths but often severely damage the internal fiber scaffold structure. In contrast, mild reagents preserve the scaffold but fail to adequately remove cells, highlighting a major challenge in chemical extraction. Therefore, the ideal preparation method should effectively eliminate the immunogenicity of the scaffolds while retaining their natural structure and original mechanical properties. Combining different approaches can leverage the strengths of each method. Additionally, developing new techniques, such as scCO_2_ processing, represents an innovative solution.

## Additional files:

***Additional Table 1:*** *Main therapies for nerve defects using nerve grafts.*

***Additional Table 2:*** *Summary of major methods for decellularization of peripheral nerves.*

***Additional Table 3:*** *Overview of clinical trials registered in ICTRP or CTG for the treatment of peripheral nerve injury using decellularized tissue.*

***Additional Table 4:*** *Comparison of selected hANGs and AVANCE clinical study reports.*

## Data Availability

*All relevant data are within the paper and its Additional files*.
